# A dual-functional antiviral and anticancer peptide to target intricate host-pathogen interacting proteins of human papillomavirus associated with head and neck cancer

**DOI:** 10.1371/journal.pone.0359513

**Published:** 2026-10-09

**Authors:** Gayathri Ashok, Soumyadip Ghosh, Sanandita Sengupta, Bikramjit Bhattacharya, Pratiksha Jana, Paul Livingstone, Sudha Ramaiah, Anand Anbarasu

**Affiliations:** 1 Department of Bio-Sciences, School of Biosciences and Technology (SBST), Vellore Institute of Technology (VIT), Vellore, Tamil Nadu, India; 2 Department of Bio-Medical Sciences, SBST, VIT, Vellore, Tamil Nadu, India; 3 School of Sports and Health Sciences, Cardiff Metropolitan University, Cardiff, United Kingdom; 4 Department of Biotechnology, SBST, VIT, Vellore, Tamil Nadu, India; Jamia Millia Islamia Central University: Jamia Millia Islamia, INDIA

## Abstract

Head and neck squamous cell carcinoma is increasingly being reported worldwide, and a major contributing factor is the incidence of human papillomavirus infections. The viral infection activates proto-oncogenes and signalling mechanisms aiding in tumorigenesis. An efficient way to block the virus-induced carcinogenesis is to identify a common medicament that can effectively inhibit the viral and host proteins. In the present study, HNSCC transcriptomic profiles and HPV genes involved in host-pathogen interactions were retrieved. The overexpressed HNSCC genes were further considered for host-pathogen and protein-protein interaction network analyses. Validated viral and host hub-genes were then docked with antiviral peptides with anticancer activities and subjected to simulation studies. Interactome analyses identified CDK1, CCNA2, CAV1, BRCA1, TERT and EGFR as host hub-genes, while E5_HPV16, E6_HPV33, E7_HPV16, 18 and 33 were screened as viral hub-genes. Functional, clinical, mutational and survival analyses filtered CDK1, CCNA2 and CAV1 as potential host therapeutic targets. Four antiviral-anticancer peptides were filtered after physico-chemical screening, which were docked with the host and viral proteins. Among them, peptide 4 (AP02569) was identified to have a high average binding affinity with an average binding energy of −1048.01 kcal/mol across all targets. The molecular dynamics simulations of peptide 4 demonstrated the overall stability among the docked complexes of all targets. It further indicated that peptide 4 had minimal fluctuation across all the docked complexes, and MM-GBSA analysis showed that peptide 4 was able to form energetically stable complexes with all target proteins. Our findings suggest that dual-functional antiviral and anticancer compounds can be potent therapeutic interventions for viral-led carcinogenesis in head and neck cancer.

## Introduction

The squamous cell carcinomas of the head and neck (HNSCC) cancer include laryngeal, hypopharyngeal, nasopharyngeal, oropharyngeal, paranasal sinus, nasal cavity, lip-oral cavity, salivary gland and metastatic squamous neck cancers. The rising incidence of these cancers as reported by the GLOBOCAN 2020, is attributed to the various risk factors such as tobacco use, alcohol consumption and occupational exposure to hazardous substances [1,2]. However, more than 70% of head and neck cancers are now related to oncogenic human papillomavirus (HPV) infection [3,4]. As per the latest GLOBOCAN statistics (2020), about 4.5% of global cancer incidence (890000 reported cases) and 4.6% of global cancer deaths (450000 reported deaths) are accounted for HNSCC. This makes HNSCC the seventh most common cancer globally [1]. A recent report on the economic burden of HNSCC revealed that the existing immunotherapy for HNSCC is likely affordable in the high-income countries, however, the risk factors associated with co-morbidities related to HNSCC such as secondary infections, decrease the cost-effectiveness in major countries [5]. Previous literatures have supported the role of high-risk HPV (HR-HPV) infections in the progression of cervical cancer [6,7]. Although several HR-HPV subtypes are reported to infect the mucosal epithelium, the prevalence of HPV 16 and 18 are reported in many cases [8]. HPV targets the undifferentiated basal cells, which are exposed as a result of the tissue disruption of the epithelial mucosa. Thereafter, the virus infects the surrounding stratified epithelia leading to a viral life cycle that is similar to that of the differentiation of the infected epithelial cell. With the increasing incidence of HNSCC, more cases are proven to be associated with HR-HPV infection [9].

The current therapeutic regimen for HNSCC consists of monoclonal antibodies such as cetuximab (against EGFR); pembrolizumab, nivolumab and chemotherapeutic cisplatin [10]. The clinical management of HNSCC has been greatly hampered by the development of resistance to therapy. Cisplatin-based chemotherapy is the current ‘gold standard’ for advanced HNSCC. However, most patients develop resistance and experience recurrence due to cisplatin’s inability to induce apoptosis in mutated cells that have an activated form of PI3K-AKT pathway and/or altered forms of DNA damage repair [11]. Mechanistically, HPV negative HNSCC contains mutated forms of TP53 tumour suppressor gene and deletions of CDKN2A gene in ~58% of patients, thus providing them with increased proliferation and survival when treated with cisplatin. Mutations or overexpression of PIK3CA, MYC, CCND1 genes or other tyrosine kinase receptors like EGFR results in the activation of the PI3K-AKT-MAPK signalling pathways for prolonged survival and chemoresistance in HPV negative cancer [11,12]. Although HPV positive HNSCC have better prognosis and better therapeutic response than HPV negative HNSCC, it is still unknown how much cisplatin contributes to the improved prognosis seen in HPV positive tumours [13]. Since cisplatin is not given alone as monotherapy, and many ongoing clinical trials are looking at deintensification regimens for HPV positive patients while also causing severe and irreversible toxicities [14]. Approximately 60−70% of all patients with recurrent or metastatic HNSCC have failed to receive some level of benefit from either pembrolizumab and nivolumab PD-1 checkpoint inhibitors used as single agent therapies and therefore indicate a significant amount of primary and acquired immune resistance [15]. Resistant to cisplatin due to TP53 mutation and PI3K-AKT activation in HPV negative HNSCC, uncertain benefits combined with extreme toxicities from cisplatin in HPV positive cancers, and extensive resistance to immunotherapies, all these limitations demonstrate a tremendous unmet medical need for novel, mechanism-based treatments for HPV associated HNSCC. While for circumventing HPV-related infections, various HPV vaccines are being administered. Although the efficiency of the HPV vaccines is not questioned, the poor rate of global administration of these vaccines implies the necessity for an alternate therapeutic strategy for HPV-HNSCC [16,17]. Antimicrobial peptides such as gramicidin, allomyrinasin, AWRK6 were key contenders for alternate therapeutics due to their selectivity and specificity [18–21]. The charge specificity of the peptides makes them more specific to cancer cells than normal cells [22,23]. The present study is designed to explore two major facets of host-pathogen interactions by (a) finding the key hub-nodes responsible for the HPI-led cancer progression using a systems biology approach and (b) screening and validating dual-functional peptides (antiviral and anticancer) against the infectious proteins for HNSCC. The target proteins and screened dual-functional peptides were studied for intermolecular interaction studies using structural bioinformatics approaches [24,25]. The antiviral-anticancer peptides, on further molecular studies, can be considered as potent therapeutics for HPV-HNSCC cancer.

Direct inhibition of viral oncoproteins such as E6/E7 has been the conventional antiviral strategy, but these proteins are intracellular, non-enzymic and interact with host partners through shallow multivalent protein-protein interfaces without a defined binding pocket, making them difficult to drug, explaining why there are no clinically approved onco-protein targeted HPV therapies [26]. However, host cell-cycle regulators & signalling cascades hijacked by these oncoproteins often function as convergent nodes within the host-pathogen interactome, sustaining both tumour-intrinsic proliferative & pro-survival programs in the tumour microenvironment regardless of continued integrity of the viral oncoproteins [27]. Therefore, identifying & co-targeting such host hub-proteins alongside the viral oncoproteins as pursued in this study offers a broader & more durable mechanistic conceptual framework for a dual functional antiviral-anti-cancer therapeutic strategy than oncoprotein inhibition alone.

## Materials and methods

### Transcriptomic profiling and differential gene expression analysis (DGEA)

HNSCC transcriptomic profiles were downloaded from The Cancer Genome Atlas (TCGA) using the *GDCquery* function of TCGAbiolinks R package [28]. A total of 520 primary tumours and 44 normal samples were considered for DGEA using DESeq2 R package. Here, the GDCquery data downloaded from TCGA was transformed into DESeq dataset, which was further analysed to obtain the differentially expressed genes (DEGs). DEGs were sorted initially according to their p-adjusted values (< 0.05) and were categorised into up and down-regulated based on the log 2-fold change (logFC). The up-regulated genes had a logFC > 1, while down-regulated genes had a logFC < −1 [29].

### Functional enrichment analysis (FEA)

The gene-set enrichment analysis was performed using the ShinyGO 0.77 web-server. The input query, consisting of up-regulated HNSCC genes with a false discovery rate (FDR) cutoff of 0.05 was subjected to redundancy removal. The input query was further analysed for KEGG biological pathways and gene ontologies (GO) namely biological processes (BP), cellular component (CC) and molecular functions (MF). The web-server analyses the input query against background genes and database and gives the significant pathways along with fold enrichment and FDR [30].

### Retrieval of HPV genes

MorCVD, a unified host-pathogen protein database was considered for the retrieval of HPV associated genes/proteins. The database has computed various kinds of interactive data such as direct interaction (DI), physical associations (PA) and other kinds of association or reactions. Currently, the database consists of 23,377 protein interactions across various human proteins for 432 unique pathogens. The users can query based on the disease, pathogen, or protein of interest. The present study retrieved HPV proteins/genes for the major oncogenic subtypes [31]. The study focused on high-risk HPV (HR-HPV) subtypes, specifically HPV 16, 18, 31, 33, and 45, which are the most clinically prevalent oncogenic subtypes reported in HNSCC. HPV 16 and 18 together account for approximately 70–90% of all HPV-positive HNSCC cases worldwide, with HPV 16 being the dominant subtype in oropharyngeal cancers [3,32]. HPV 33 and 31 also contribute to a smaller but documented proportion of HNSCC. HPV 45 was not retrieved because no interaction data were available for HPV 45 in the MorCVD database for the gene set identified in this study, and its reported prevalence in HNSCC is substantially lower than HPV 16 and 18. The host genes associated to the HPV subtypes were then cross-validated with the HNSCC transcriptomic profile to screen the up-regulated HNSCC genes and its corresponding interacting pathogen genes were considered for further analyses.

### Host-pathogen interaction data curation

Host-pathogen interactome was constructed using PredHPI, an integrated web-server platform hosting HPI based on sequences. The interaction data of the finalised host and pathogen proteins was retrieved from PredHPI, which relied upon HPIDB, MINT, DIP, BioGRID, IntAct, VirHostNet, STRING, VIH-1 NCBI, ArbaHPI, PHISTO databases to curate the interactions. The interaction was considered for the e-value of 1e^-10^ with a minimum identity of 30% and coverage of 60%, which is the default setting for the server [33].

### Network construction and analysis

To understand the interactions among the identified HNSCC genes, we employed STRING database. The interacting host proteins were filtered at a medium confidence score of>0.4 and searched through various active interactive sources such as textmining, experimentally derived data, database, co-expression, homology, gene fusion and co-occurrence [34]. Thereafter, both the interaction data were compiled for further analyses. The HPI-PPI was visualised using Cytoscape [35]. cytoHubba plugin was used to filter the hub-nodes based on topological metrics such as betweenness centrality, degree and closeness centrality. These parameters sort the hub-nodes based on the influence of the nodes on other nodes, direct number of interactors and closeness of the nodes in the interactome, respectively. The hub-nodes were selected separately for host and pathogen, based on the average topological metrics [36,37].

### Functional, clinical and survival profiling of key genes

The clinical and functional profiling of the host genes were then performed to screen the functionally significant genes for HPV-positive HNSCC. Overall survival was estimated for the host genes using the Kaplan-Meier plot [38]. The hazard ratio (HR), median overall survival for low and high groups were analysed for the hub-nodes. The mutational landscape of the identified host-hub genes were studied across the clinical samples of head and neck cancer using the cBioportal for genomics portal [39]. Thereafter, the median expression of the hub-nodes was cross-validated across grades (1 -4) and also in HPV positive and negative samples using UALCAN [40].

### Host-Pathogen protein structure validation

Filtered target host proteins were then retrieved from experimentally derived structures of RSCB-protein databank (PDB) and Alphafold database. The Alphafold database employs a neural network algorithm to predict protein structures with precise atomic configuration. The database was validated by the Critical Assessment of protein Structure Prediction (CASP14) [41,42]. The pathogen proteins were modelled by *ab initio* modelling using Robetta [43].The targets were then subjected to energy minimisation employing Steepest Descent by a 2000 steps process involving conjugate gradient algorithms using Swiss PDBViewer [44]. Thereafter, all the targets were checked for Ramachandran favoured regions using ProCheck before further analyses [45].

### Antiviral peptide screening

Antiviral peptides were screened from the antimicrobial peptide database (APD3) currently updated as APD6. The public repository hosts over 2500 natural peptides across various species (as accessed on 10^th^ April, 2024). As of 1^st^ January, 2026, the APD6 contained 3379 natural AMPs. The peptides can be classified into antibacterial, antibiofilm, anti-endotoxin, antiviral, anti-HIV, anticancer, and anti-inflammatory, among the various types. The peptides deposited on the repository with unique accession IDs were provided with the sequence, source, length, net charge, hydrophobic residues, Boman index and potential activity [46].

### Physico-chemical property analysis of screened peptides

After the initial screening of the peptides based on charge (≥2) and hydrophobic residues (15–50%), the toxic peptides were filtered out by a deep-learning process using ToxlBTL server [47]. Stable and cell penetrating peptides were then filtered using ProtParam and BChemRF-CP servers, respectively [48,49]. The cell penetrability was evaluated using a machine learning algorithm that differentiated the cell penetrating ones from the non-cell penetrating peptides. Thereafter, peptides were checked for anti-inflammatory, antigenicity and hemolytic properties. The screened peptides were then subjected to a machine learning-based anticancer activity prediction using MLACP 2.0. The server based on a convolutional neural network predicts the anticancer activity for peptides according to the non-redundant training set based on literature and database search and evaluates 17 different features making the results more accurate [50].

### Peptide modelling and validation

The filtered peptides were then modelled using PEP-FOLD3, which uses a faster *de novo* structure prediction approach for peptide modelling. The server is an updated version of PEP-FOLD, with the option of modelling bias peptide conformational sampling by providing a reference structure. The modelling depends on the structural alphabet concept, where the peptides are considered as fragments with specific descriptors predicted using the Hidden Markov Models algorithm [51].The modelled peptides were then subjected to energy minimisation as previously explained and Ramachandran favoured regions were detected across all the models. The energy-minimised peptides were then used for further docking analysis.

### Intermolecular interactions analysis

The intermolecular docking among the screened peptides and target host and pathogen proteins were identified using the ClusPro docking server. The ClusPro algorithm employs rigid body docking based on fast fourier transform correlation techniques. With a grid box of 128 Å x 128 Å x 128 Å and spacing of 1 Å, the algorithm retains around 20000 structures with best surface complementarity scores, which undergo further free energy filtering. Thereafter, energetically favourable structures were clustered based on root mean square deviation (RMSD). The ligands with the maximum number of neighbours within the selected clustering radius were then listed and the top models were filtered [52]. The ClusPro Score is a weighted summation of van der Waal’s, electrostatic, and desolvation energies calculated on an individual basis for all atoms within the whole complex. The scoring function that calculates this (the ClusPro Score) is part of ClusPro’s PIPER FFT search algorithm and thus will be displayed in the standard output as the total interaction energy. Larger negative values indicate better predicted complementarity. The intermolecular interactions compiling the canonical and non-canonical interactions were visualised using LigPlot [53]. For the ClusPro protein-peptide docking mode used in this study, the default clustering radius was set, and cluster representatives were selected on the basis of cluster size, that is, the model possessing the maximum number of neighbouring structures within the clustering radius was designated as the top-ranked pose for each protein-peptide complex. Based on the docking results, the lead molecule along with two control peptides (Although there are no approved therapeutic peptides are available against the selected target). Based on the literature survey, we selected two control peptides as references, which have some evidence and have been at least experimentally validated for a specific target, has further taken forward for validatory docking using the HDOCK server, an independent FFT-based global docking algorithm that has been benchmarked against ClusPro [54,55].

### Molecular dynamics simulation

All-atom molecular dynamics (MD) simulations of the protein-peptide complexes were conducted using GROningen MAchine for Chemical Simulations (GROMACS) version 2024.2 [56]. To evaluate the structural stability and interaction dynamics, each host and HPV apoprotein and all the protein-peptide complexes were simulated for 100,000 ps (100 ns). The protein topology was generated using the CHARMM36-Jul2022 force field in conjunction with the TIP3P water model [57]. The solvated system was prepared by placing the complex at the center of a cubic box with a uniform 1.0 nm buffer distance from the box edges, followed by solvation with water molecules and neutralisation using appropriate concentrations of Na⁺and Cl^−^ ions. Energy minimisation was performed using the steepest descent algorithm for 50,000 steps by applying a maximum force convergence criterion of 1000 kJ/mol/nm. Subsequently, the system underwent two-phase equilibration under canonical (NVT) and isothermal–isobaric (NPT) ensembles. Both equilibration phases were executed for 100 ps using the leap-frog integrator. The NVT phase employed a modified Berendsen thermostat to maintain the system temperature at 300 K, and the velocities were assigned according to a Maxwell distribution. The NPT phase used a Parrinello–Rahman barostat to maintain a pressure of 1 bar. Long-range electrostatic interactions were handled via the Particle Mesh Ewald (PME) method with a Fourier spacing of 0.16 nm and a fourth-order (cubic) interpolation scheme. The production MD simulation was then performed for 100 ns with a time step of 2 fs. MD simulations were performed of all the complexes in duplicate with different initial velocities to adequately sample the equilibrium state and confirm the absolute stability of the inhibitor complexes. Post-simulation trajectory analyses were conducted to assess the dynamic and structural stability of both the apoprotein and docked complexes. The parameters analysed included root mean square deviation (RMSD), root mean square fluctuation (RMSF), radius of gyration (Rg), solvent-accessible surface area (SASA), hydrogen bond analysis, and Lennard-Jones short-range interaction energy (LJ-SR), which represent van der Waals forces [58]. Visualisation and graphical representation of trajectory data were performed using QtGrace software version 0.2.6.

### MM-GBSA analysis for binding free energy calculation

The gmx_MMPBSA tool was used to determine binding free energy values for the protein-peptide complexes investigated in this study [59,60]. The gmx_MMPBSA tool uses the Molecular Mechanics Generalised Born Surface Area (MM-GBSA) method to provide estimates of the binding free energy of peptides to target proteins. In addition to validating the MD simulations, these free energy values provided a quantitative assessment of the binding affinities of peptide 4 to a number of the host and HPV target proteins studied. The MM-GBSA method calculates the total free energy of binding of a protein-protein complex from the MD simulation trajectory data by using Generalized Born (GB) solvation models. By default, all calculations were run with consistent default settings for both the parameter settings and the temperature of 298.15 K to enhance the ability to reproduce and compare the results of the studies presented here. The igb = 5 setting was also used to configure the solvation model to correctly calculate the implicit solvent effects as approximated through the use of the GB method. The dielectric constants were set to their typical values of 1.0 for the internal dielectric constant and 78.5 for the external dielectric constant to accurately compute the solvation energies and electrostatic contributions to the binding affinity. The results obtained from the application of the MM-GBSA method to each of the protein-peptide complexes presented a comprehensive view of the energetics of protein-peptide interactions, including the binding free energy values calculated for each of the different protein-peptide combinations that were studied. The results illustrated the variation in the binding free energies for the different protein-peptide complexes that were studied and helped elucidate the dynamic molecular interactions involved. To determine amino acids that are major contributors to the binding free energies in all the target protein-peptide 4 complexes, we performed per-residue energy decomposition analysis with the gmx_mmpbsa. This tool breaks down the MM-GBSA (molecular mechanics generalized born surface area) energy into four separate components such as van der Waals, electrostatic, polar solvation and nonpolar solvation. We did this breakdown on the entire length of all the target protein-peptide 4 complexes’ MD simulations that ran over 100 ns. It calculates individual residue-level energetic contributions encompassing van der Waals, electrostatic, polar solvation, and non-polar solvation energy terms. Those residues that contribute the most negative energy per residue will be considered as those driving the stabilisation of the complex through interaction at their respective binding interfaces. Energy component visualisation and interpretation of the results were achieved using the analysis tools available in gmx_MMPBSA_ana. An overall schematic depiction of the *in silico* pipeline is represented in Fig 1.

**Fig 1:**
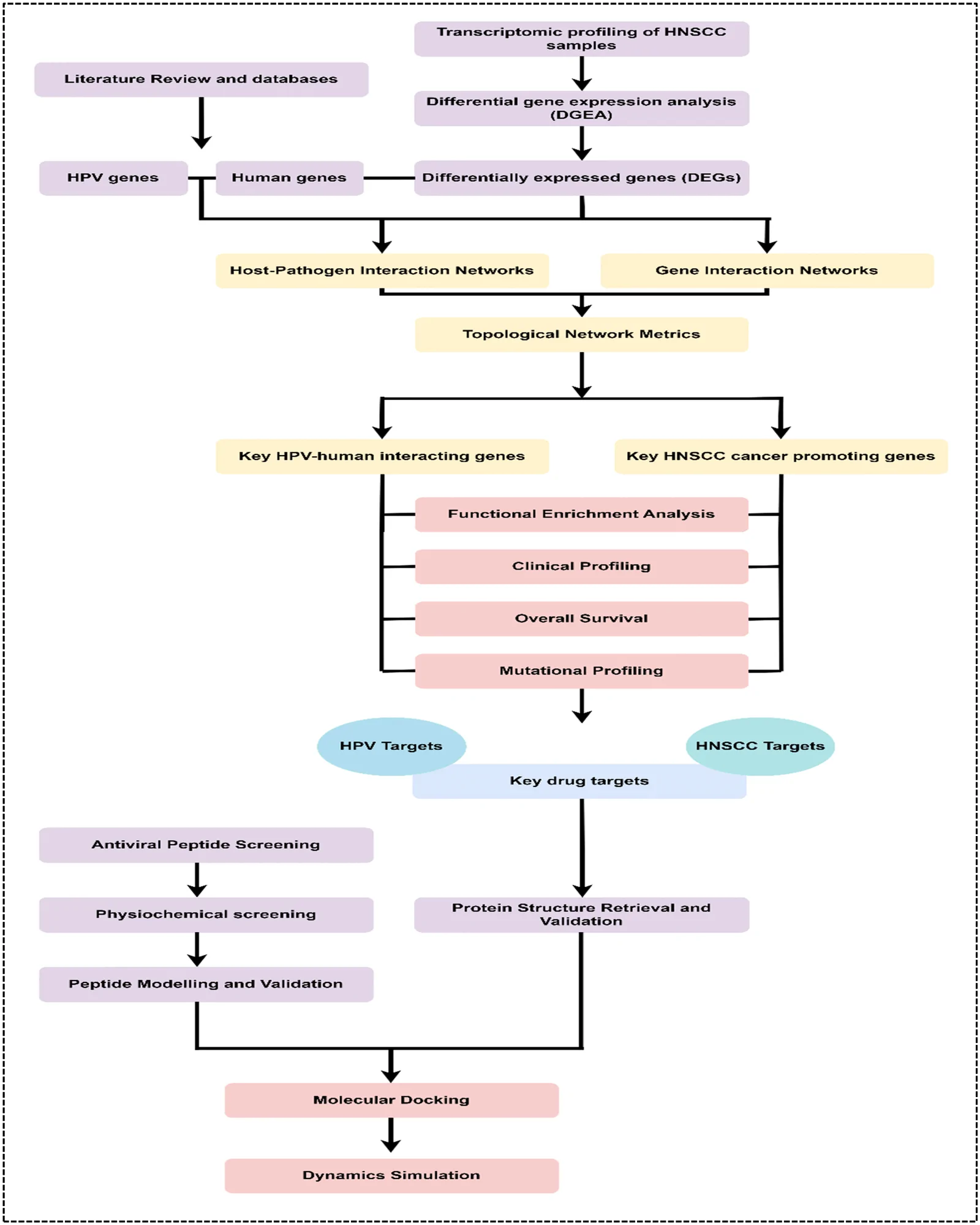
Schematic workflow depicting the *in silico* pipeline employed for the study.

## Results

### Differentially expressed genes of HNSCC

DGEA resulted in 21229 statistically significant DEGs, which were further categorised into up and down-regulated based on the logFC values. Around 4166 genes were identified to be up-regulated with a logFC > 1 and p-adj < 0.05, while 3001 genes were down-regulated with a logFC < −1.

### Functional enrichment analysis

The up-regulated genes on FEA highlighted key biological pathways with most number of genes (nG) involved to be pathways in cancer (nG: 82; Fold Enrichment (FE): 1.72), neuroactive ligand-receptor interaction (nG: 75; FE: 2.39), human papillomavirus infection (nG: 66; FE: 2.22), PI3K-Akt signaling pathway (nG: 62; FE: 1.95), cytokine-cytokine receptor interaction (nG: 60; F: 2.27), neutrophil extracellular trap formation (nG: 56, FE: 3.29), alcoholism (nG: 50; FE: 2.98), systemic lupus erythematosus (nG: 47; FE: 3.85), focal adhesion (nG: 43; FE: 2.40) and viral carcinogenesis (nG: 42; FE: 2.30). The Fig 2a depicts the major KEGG pathways according to fold enrichment. The complete list of pathways is given in S1 Table.

**Fig 2:**
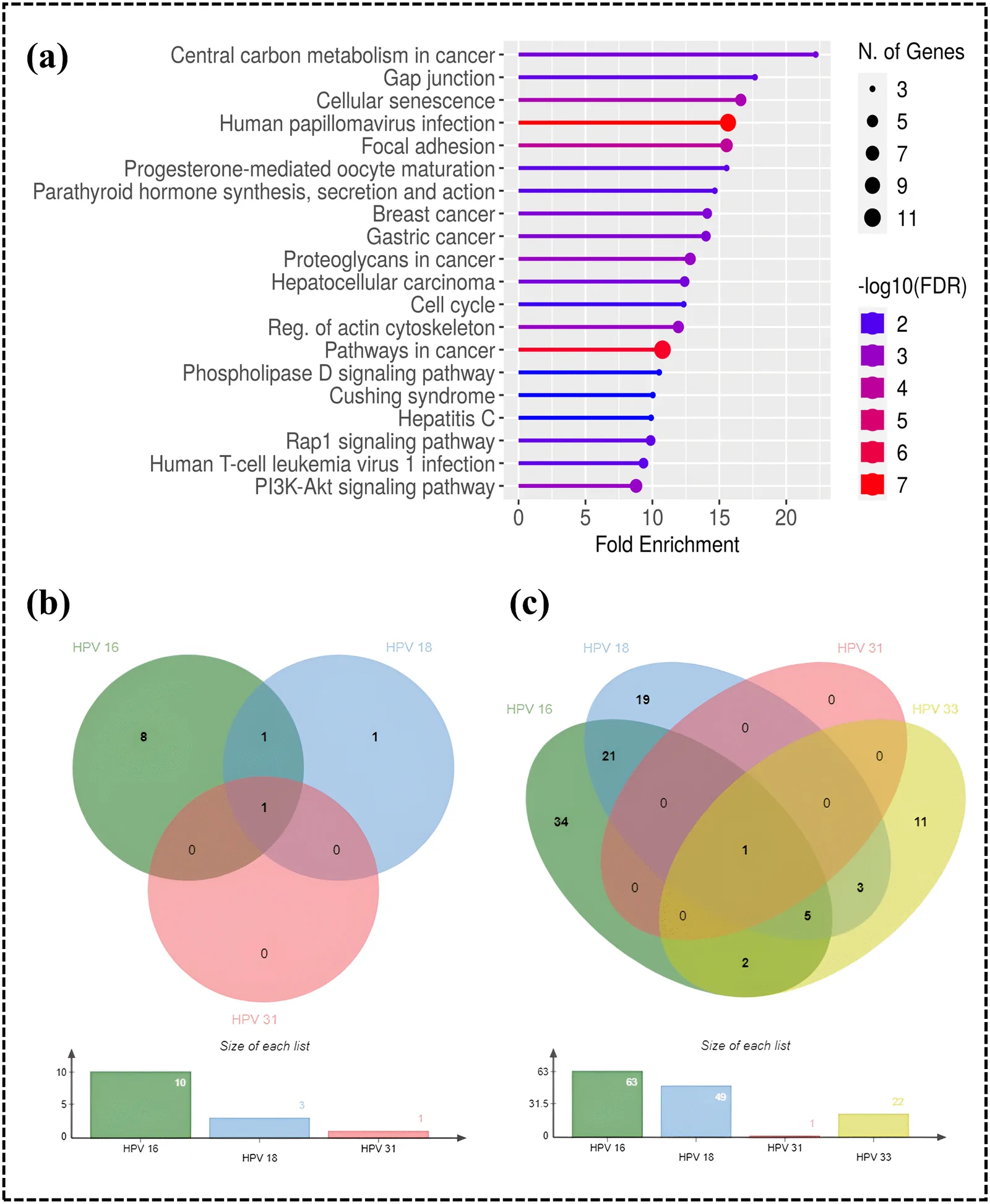
(a) Functionally significant pathways enriched by HNSCC over-expressed genes (b) Direct association among the subtypes of HPV (c) Physical association among the subtypes of HPV.

### HNSCCC-HPV gene association

Pathogen-host proteins/genes retrieval on the different HPV subtypes yielded 45 direct interactions DI and 325 PA with HPV 16; 16 DI and 308 PA with HPV 18; 8 DI and 9 PA with HPV 31; 116 PA with HPV 33 and one PA with HPV 35. Thereafter, we checked the expression profile of the host genes and filtered those associations with up-regulated HNSCC genes resulting in 10 DI and 63 PA for HPV 16; three DI and 49 PA for HPV 18; one DI and PA for HPV 31 and 22 PA for HPV 33 (Fig 2b-c). Around 15 pathogen genes across HPV 16, 18 and 33 subtypes and 48 host genes up-regulated in HNSCC were filtered for further analyses (S2 and S3 Tables).

### HPI network of HNSCCC-HPV genes

The host-pathogen interaction data was curated for the filtered pathogen and host genes across databases such as HPIDB, MINT, DIP, BioGRID, IntAct, VirHostNet, STRING, VIH-1 NCBI, ArabHPI, PHISTO and alignment options consisting of 30% identity with a coverage of 60% and 30% for host and pathogen proteins respectively. Further, we also curated the protein-protein interaction data for the host proteins and their first set of interactors, resulting in a network of average node degree of 4.49 and PPI enrichment p-value of 9.78e-09. Thereafter, the HPI and PPI interactome were constructed for further analyses.

### Topological network metrics evaluation

A complete interactome of the host and pathogen genes is provided in Fig 3a. Topological metrics namely betweenness centrality (BC), degree (DG) and closeness centrality (CC) were computed for each node (host and pathogen). Key hub-genes were filtered separately for host and pathogen genes to identify the central nodes responsible for the host-pathogen interaction. After categorising the top 10 genes according to BC, DG and CC, genes namely, EGFR (476.34), BRCA1 (207.14), CDK1 (162.86), CAV1 (142.48), TERT (130.492) and CCNA2 (100.13) were the top hub-genes common in all three parameters. Among the pathogen genes, E5_HPV16, E7_HPV33, E7_HPV16, E7_HPV18 and E6_HPV33 computed high metrics, with E5_HPV16 showing an average of 398.52, followed by E7_HPV33, E7_HPV16, E7_HPV18 and E6_HPV33 with an average of 213.63, 213.63, 193.21 and 139.94 respectively (Table 1 and Fig 3b). The interactions among the host and pathogen hub-genes were also studied, highlighting EGFR and CAV1 to be directly interacting with E5_HPV16; TERT interacting directly with E6_HPV33; BRCA1 interacting with E6_HPV33, E7 gene of HPV16, 18 and 33. Similarly, CDK1 and CCNA2 were directly interacting with E7 gene of HPV16, 18 and 33 (Fig 3c).

**Table 1:** CytoHubba identified HPV and host hub-genes.

Hub-Genes	GENE	BETWEENNESS	DEGREE	CLOSENESS	AVERAGE
Host	EGFR	1336.08	47	45.95	476.34
	CDK1	415.85	30	42.75	162.87
	BRCA1	548.02	29	44.42	207.15
	CCNA2	235.16	24	41.25	100.14
	TERT	329.06	23	39.42	130.49
	CAV1	369.78	21	36.68	142.49
Pathogen (HPV)	E7_HPV33	572.38	24	44.53	213.64
	E7_HPV16	572.38	24	44.53	213.64
	E7_HPV18	512.79	23	43.86	193.22
	E5_HPV16	1138.96	16	40.62	398.52
	E6_HPV33	368.84	13	38	139.95

**Fig 3:**
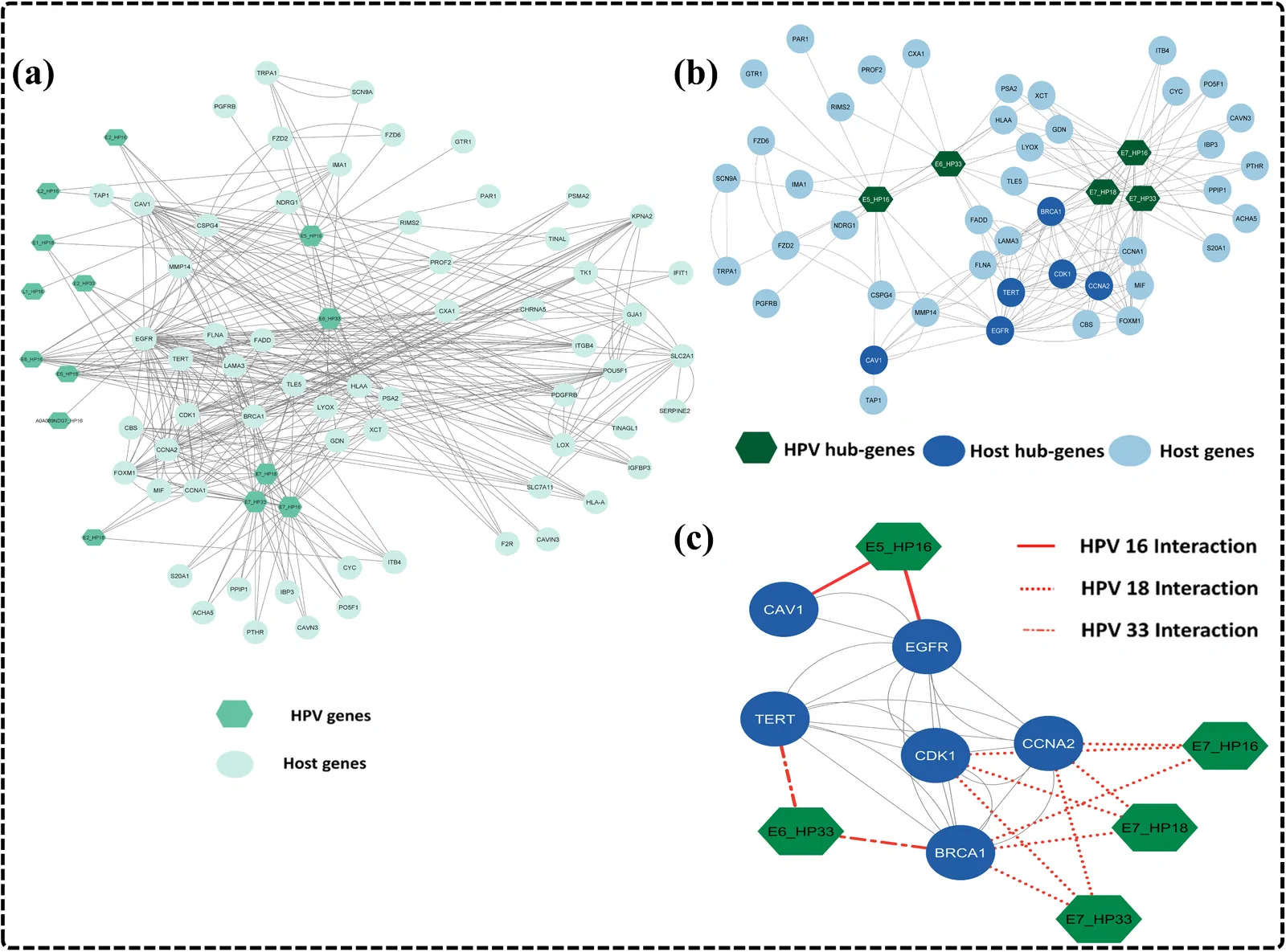
Host-Pathogen Interactome (a) Overall HPV-host interactome (b) HPV and host hub-genes and its interacting host genes (c) Interactions among HPV hub-genes and host hub-genesgenes.

### Functional and clinical relevance of key host genes

Functional role of the host-hub-genes were then cross-validated identifying EGFR, CCNA2 and TERT to be enriched in human papillomavirus infection (hsa05165). Interestingly, the major pathways enriched by the overall HNSCC genes such as focal adhesion, pathways in cancer, PI3K-Akt signalling and viral carcinogenesis were also the key functional roles of the identified hub-genes (Table 2). This indicates the crucial role of the identified hub-genes for HNSCC progression. Additionally, the direct or physical association of these genes with the viral oncogenes further validate the results of the study. Clinical survival rate of the genes further indicated the critical nature of the over-expressed genes for HNSCC. Among the hub-genes, CCNA2, CDK1, CAV1 and EGFR showed high HR and critical median overall survival (MOS) for high expression cohort (Fig 4a). CDK1 had MOS_high_ of 47.67 months while MOS_low_ of 90.57 months (for CDK1, HR = 1.4 logrank P = 0.038). Similarly, CCNA2 showed MOS_high_ of 42.97 months when compared to MOS_low_ of 90.1 months (for CCNA2, HR = 1.43 logrank P = 0.016); CAV1 had MOS_high_ of 32.93 months while MOS_low_ of 58.73 months (for CAV1, HR = 1.53 logrank P = 0.0024). and EGFR with a MOS_high_ of 42.97 months and MOS_low_ of 58.27 months (for EGFR, HR = 1.42 logrank P = 0.014). BRCA1 and TERT with a low HR of 0.64 and high MOS_high_ of 69.43 and 110.47 months, respectively were not considered for further analyses. Mutational profiling of the hub-genes across 636 clinical cases further identified EGFR with 11% mutations while CAV1, CCNA2 and CDK1 showed<1% mutations indicating a more stable therapeutic target for HNSCC (Fig 4b). The expression profile of the identified hub-genes in HPV associated HNSCC cases depicted CDK1, CCNA2, BRCA1 and TERT to be over-expressed in HPV positive cases with a median expression of 83.13, 38.41, 18.17 and 0.21 (Fig 4c). However, all the hub-genes showed increased expression across various stages (S1–S4) for HNSCC. Based on the functional, clinical, mutational and survival plot analyses, CDK1, CCNA2 and CAV1 were considered as potent therapeutic targets for further analyses.

**Table 2:** Hub-genes enriched functionally significant KEGG pathways.

Hub-Genes	KEGG ID	KEGG Pathways	Gene count	FDR	GENE INVOLVED
EGFR	hsa05165	Human papillomavirus infection	12	2.32E-08	TNFRSF1A, ITGB4, CCNA1, PDGFRB, EGFR, FADD, TERT, FZD2, LAMA3, FZD6, HLA-A, CCNA2
	hsa05200	Pathways in cancer	11	2.00E-05	CCNA1, PDGFRB, EGFR, FADD, TERT, F2R, FZD2, LAMA3, FZD6, SLC2A1, CCNA2
	hsa04510	Focal adhesion	6	0.0016	ITGB4, PDGFRB, EGFR, LAMA3, CAV1, FLNA
	hsa05205	Proteoglycans in cancer	6	0.0016	DCN, EGFR, FZD2, CAV1, FZD6, FLNA
	hsa04540	Gap junction	4	0.0039	PDGFRB, EGFR, GJA1, CDK1
	hsa05163	Human cytomegalovirus infection	5	0.0089	TNFRSF1A, EGFR, FADD, TAP1, HLA-A
	hsa04151	PI3K-Akt signaling pathway	6	0.0094	ITGB4, PDGFRB, EGFR, F2R, LAMA3, BRCA1
	hsa05224	Breast cancer	4	0.0168	EGFR, FZD2, FZD6, BRCA1
	hsa05226	Gastric cancer	4	0.0168	EGFR, TERT, FZD2, FZD6
	hsa05160	Hepatitis C	4	0.018	TNFRSF1A, EGFR, FADD, IFIT1
	hsa05225	Hepatocellular carcinoma	4	0.018	EGFR, TERT, FZD2, FZD6
	hsa05230	Central carbon metabolism in cancer	3	0.018	PDGFRB, EGFR, SLC2A1
	hsa04810	Regulation of actin cytoskeleton	4	0.0389	ITGB4, PDGFRB, EGFR, F2R
	hsa04928	Parathyroid hormone synthesis, secretion and action	3	0.0434	EGFR, MMP14, PTHLH
CDK1	hsa04218	Cellular senescence	5	0.0025	CCNA1, IGFBP3, CDK1, HLA-A, CCNA2
	hsa04540	Gap junction	4	0.0039	PDGFRB, EGFR, GJA1, CDK1
	hsa05170	Human immunodeficiency virus 1 infection	5	0.0079	TNFRSF1A, FADD, TAP1, CDK1, HLA-A
	hsa05203	Viral carcinogenesis	4	0.0266	CCNA1, CDK1, HLA-A, CCNA2
	hsa04914	Progesterone-mediated oocyte maturation	3	0.0371	CCNA1, CDK1, CCNA2
BRCA1	hsa04151	PI3K-Akt signaling pathway	6	0.0094	ITGB4, PDGFRB, EGFR, F2R, LAMA3, BRCA1
	hsa05224	Breast cancer	4	0.0168	EGFR, FZD2, FZD6, BRCA1
CCNA2	hsa05165	Human papillomavirus infection	12	2.32E-08	TNFRSF1A, ITGB4, CCNA1, PDGFRB, EGFR, FADD, TERT, FZD2, LAMA3, FZD6, HLA-A, CCNA2
	hsa05200	Pathways in cancer	11	2.00E-05	CCNA1, PDGFRB, EGFR, FADD, TERT, F2R, FZD2, LAMA3, FZD6, SLC2A1, CCNA2
	hsa05166	Human T-cell leukemia virus 1 infection	6	0.0016	TNFRSF1A, CCNA1, TERT, HLA-A, SLC2A1, CCNA2
	hsa05169	Epstein-Barr virus infection	6	0.0016	PSMC4, CCNA1, FADD, TAP1, HLA-A, CCNA2
	hsa04218	Cellular senescence	5	0.0025	CCNA1, IGFBP3, CDK1, HLA-A, CCNA2
	hsa05203	Viral carcinogenesis	4	0.0266	CCNA1, CDK1, HLA-A, CCNA2
	hsa04914	Progesterone-mediated oocyte maturation	3	0.0371	CCNA1, CDK1, CCNA2
TERT	hsa05165	Human papillomavirus infection	12	2.32E-08	TNFRSF1A, ITGB4, CCNA1, PDGFRB, EGFR, FADD, TERT, FZD2, LAMA3, FZD6, HLA-A, CCNA2
	hsa05200	Pathways in cancer	11	2.00E-05	CCNA1, PDGFRB, EGFR, FADD, TERT, F2R, FZD2, LAMA3, FZD6, SLC2A1, CCNA2
	hsa05166	Human T-cell leukemia virus 1 infection	6	0.0016	TNFRSF1A, CCNA1, TERT, HLA-A, SLC2A1, CCNA2
	hsa05226	Gastric cancer	4	0.0168	EGFR, TERT, FZD2, FZD6
	hsa05225	Hepatocellular carcinoma	4	0.018	EGFR, TERT, FZD2, FZD6
CAV1	hsa04510	Focal adhesion	6	0.0016	ITGB4, PDGFRB, EGFR, LAMA3, CAV1, FLNA
	hsa05205	Proteoglycans in cancer	6	0.0016	DCN, EGFR, FZD2, CAV1, FZD6, FLNA

**Fig 4:**
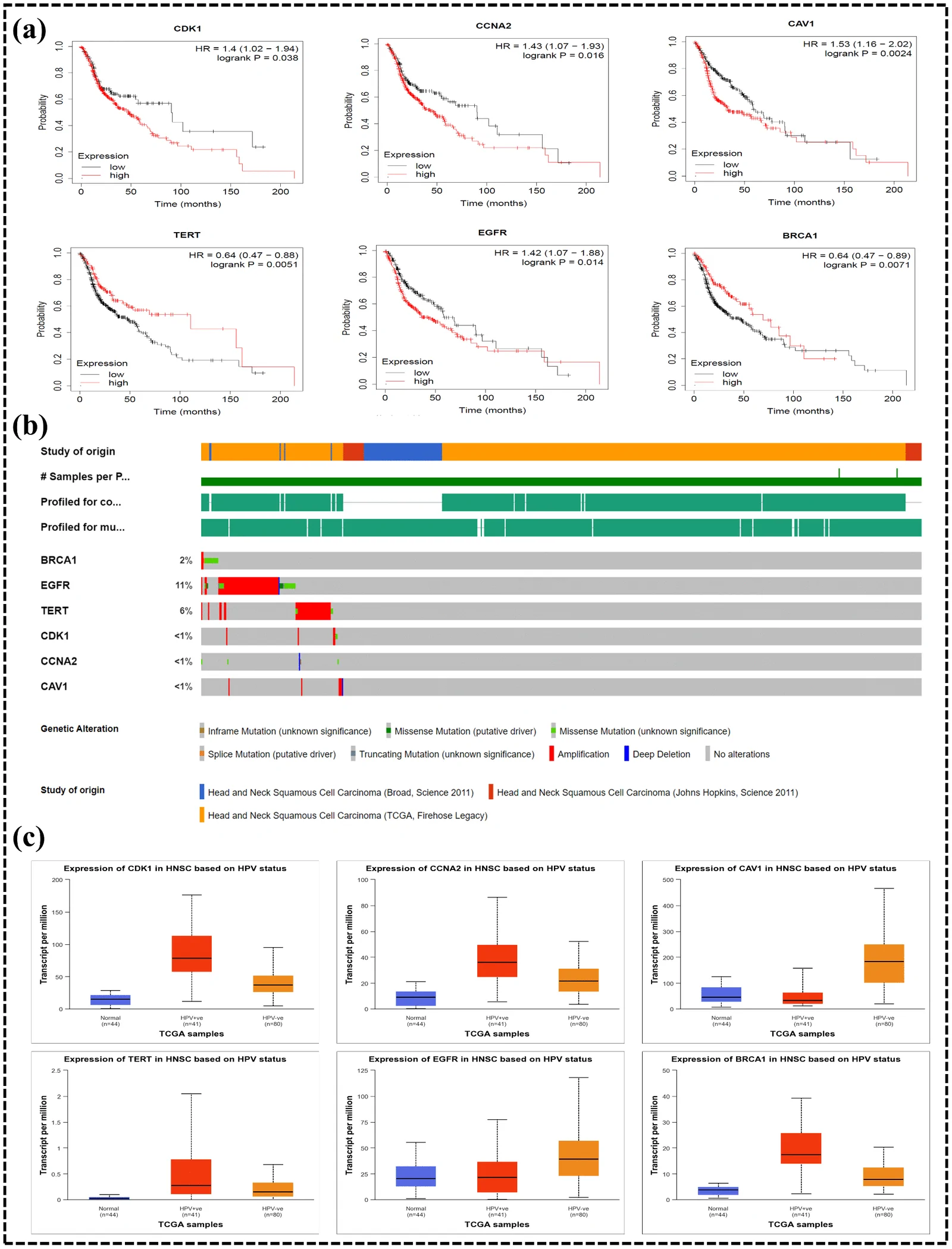
Hub-gene validation (a) Kaplan Meier plot depicting overall survival of the identified host hub-genes (b) Oncoprint representing the mutational landscape of the host hub-genes in head and neck cancers (c) Comparative expression profile of the hub-genes in HPV positive and negative samples.

### Protein structure validation studies

The protein structures of the target host proteins were then retrieved from PDB and Alphafold database. CAV1 protein structure with an accession id of AF-Q03135-F1, CDK1 with an accession ID of AF-P06493-F1 and CCNA2 structure with a PDB ID of 4EOJ were selected for the study. Thereafter, energy minimisation was performed on the protein structures, which resulted in the minimisation of initial energy (E_i_) of −4698.77 kJ/mol (CAV1), −8490.57 kJ/mol (CCNA2) and −13087 kJ/mol (CDK1) to final energy (E_f_) −6367.92 kJ/mol, −12029 kJ/mol and −15708 kJ/mol respectively. The Ramachandran favoured regions of the three target proteins were also analysed indicating>98% for all the models (S4 Table). The host proteins identified were then modelled using Robetta server, resulting in modelled structure with a confidence score>0.6 for further studies. The pathogen target proteins were similarly subjected to energy minimizations from an E_i_ of −2020.04 kJ/mol (E5_HPV16), −2011.33 kJ/mol (E7_HPV16), −3506.62 kJ/mol (E7_HPV18), −3035.22 kJ/mol (E7_HPV33) and −7939.59 kJ/mol (E6_PHV33) to a E_f_ of −3435.75 kJ/mol, −3322.72 kJ/mol, −4997.89 kJ/mol, −4272.62 kJ/mol and −10209.13 kJ/mol respectively. All the structures showed >98% Ramachandran favoured regions and were considered for further docking studies (S5 Table)

### Screened potent AVP-ACP, modelling and validation

Around 207 AVP (As of 10 April, 2024) were screened for the present study, which were initially screened based on net charge ≥2, resulting in 161 peptides. Thereafter, we filtered the peptides based on 15–50% hydrophobic residues. The 102 peptides were then filtered based on toxicity and 60 non-toxic peptides were then analysed for stability. Around 44 stable peptides were then investigated for their cell penetrability, which led to 9 peptides and then those 9 peptides were further assessed for hemolytic, anti-inflammatory, non-allergic and anticancer activity. Among the nine peptides, only four non-hemolytic, anti-inflammatory, non-allergic and anticancer peptides (Complete screening process presented in S6 Table) with accession IDs, namely, AP00677, AP00355, AP01010 and AP02569, were screened for further analyses and are listed in Table 3. The screened peptides were then modelled and the resultant structures were characterised for their secondary structure and further validation through Ramachandran favoured regions (S7 Table).

**Table 3:** Summary of antiviral peptides after physico-chemical screening.

Peptides	APD ID	Sequence	ACP/non-ACP	Net Charge	Hydrophobic Residue%	Toxicity	Half-Life	Instability Index	Cell Penetration
Peptide_1	AP00677	GLRKKFRKTRKRIQKLGRKIGKTGRKVWKAWREYGQIPYPCRI	ACP (0.84)	16	27	non-toxic	30 hrs	Stable (10.43)	CPP (85.78%)
Peptide_2	AP00355	ANTAFVSSAHNTQKIPAGAPFNRNLRAMLADLRQNAAFAG	ACP (0.673)	3	47	non-toxic	4.4 hrs	stable (37.12)	CPP (52.29%)
Peptide_3	AP01010	SMWSGMWRRKLKKLRNALKKKLKGE	ACP (0.747)	9	36	non-toxic	1.9 hrs	stable (18.36)	CPP (56.00%)
Peptide_4	AP02569	KFFKRLLKSVRRAVKKFRKKPRLIGLSTLL	ACP (0.924)	12	43	non toxic	1.3 hrs	stable (25.8)	CPP (88.52%)

### Intermolecular interactions assessment

The intermolecular interactions among the peptides and the target proteins were assessed, revealing peptide 4 to have highest BE of −1048.012 kcal/mol across all the targets. This was followed by peptide_1, peptide_2 and peptide_3 with an average BE of −1032.6 kcal/mol, −966.125 kcal/mol and −934.625 kcal/mol (Table 4).

**Table 4:** Intermolecular docking analysis on HPV and host proteins with the screened antiviral peptides.

Target proteins		Binding Energy (kcal/mol)			
		Peptide 1	Peptide 2	Peptide 3	Peptide 4
Pathogen (HPV) proteins	E5_HPV16	−1099.5	−1097.8	−947.5	**−1118.7**
	E6_HPV33	−884	**−1007.5**	−799.9	−901.7
	E7_HPV16	−1112.2	−933.9	−1041.7	**−1202.3**
	E7_HPV18	**−1021.4**	−855.2	−848.6	−950.3
	E7_HPV33	−1059.3	−981.2	−893.4	**−1071.3**
Host proteins	CAV1	−1066.7	−1034.9	−1050.1	**−1166.2**
	CCNA2	**−1117.4**	−907.2	−984.5	−1085.8
	CDK1	−900.3	**−911.3**	−911.3	−887.8
**Average BE**		−1032.6	−966.125	−934.625	**−1048.012**

Peptide_4 on complex with the viral target proteins namely E5_HPV16, E7_HPV16 and E7_HPV33 showed comparatively higher binding affinity with a BE of −1118.7 kcal/mol, −1202.3 kcal/mol and −1071.3 kcal/mol respectively. Among the human targets, peptide_4 showed good binding affinity with CAV1 (BE: −1166.2 kcal/mol), CDK1 (−887.8 kcal/mol) and CCNA2 (−1085.8 kcal/mol). The binding affinity of the docked complexes can be attributed to the hydrogen bonds and other non-canonical interactions established between them. Docked complex of CAV1-peptide four showed 10 H-bonds and hydrophobic interactions among 13 CAV1 residues and 12 peptide residues (Fig 5a). In case of CCNA2, the docked complexes exhibited 12 H-bonds, one salt-bridge and hydrophobic interaction among nine CCNA2 and eight peptide residues (Fig 5b), while CDK1-peptide docked complex had seven H-bonds; two salt bridges and hydrophobic interactions among 14 CDK1 and 12 peptide residues. The H-bond in the active site (R127) of CDK1 was also highlighted during the analysis (Fig 5c). The docked complexes of the peptide 4 with viral targets exhibited strong intermolecular interactions among them thereby validating the high binding affinity. For the docked complex with E5_HPV16 protein, three H-bonds and hydrophobic interactions among 18 E5 protein and 11 peptide residues were established (Fig 6a). Conversely, E6_HPV33 depicted eight H-bonds, one salt bridge and hydrophobic interactions among 20 E6 protein residues and 11 peptide residues (Fig 6b). The oncogenic E7 protein in HPV16, 18 and 33 exhibited 16 (Fig 6c), 14 (Fig 7a) and 12 H-bonds (Fig 7b), respectively with peptide 4. To further validate the docking-based ranking of the lead peptide, peptide 4 was independently re-docked against all eight host and HPV target proteins using the HDOCK server, alongside two reported reference peptides, Pep11** (seq: KEKEEYNSNCSCIACIGLI) and Pep-7 (seq: SACPKGLDWCGG), originally characterised against E6_HPV16 and E7_HPV16, respectively [61–63]. Although no experimentally validated peptide inhibitors have been reported to date for selected target proteins such as CAV1, CCNA2, CDK1, E5_HPV16, E6_HPV33, E7_HPV18 and E7_HPV33 except E7_HPV16. However, HPV33 E6 belongs to the high-risk α-9 group and shares substantial structural similarity with HPV16 E6. So, therefore, we used Pep11** and Pep-7, which were docked against all the target proteins for comparative benchmarking. The HDOCK docking and confidence scores for peptide 4 and the two control peptides across all targets are presented in S8 Table. Peptide 4 consistently outperformed both reference peptides across all eight targets, yielding markedly more favourable HDOCK docking scores, most notably against CAV1 (−330.32) and E5_HPV16 (−275.05), compared with Pep11** (−203.59 and −181.37) and Pep-7 (−169.26 and −181.89) for the same targets. The docking score in the HDOCK server for protein-protein docking, is a dimensionless empirical score rather than a specific physical unit. It is calculated using knowledge-based iterative scoring functions. A more negative docking score indicates a more stable and favourable binding model. Confidence scores for peptide 4 were also higher than for either reference peptide across all targets, corroborating the binding preference observed in the ClusPro-based docking analysis and supporting peptide 4 as the lead dual-functional candidate among the screened peptides. As peptide 4 was revealed to have the highest docking score and the lowest BE based on docking and exhibited strong intermolecular interactions across all the targets, further proceeding with MD simulations.

**Fig 5:**
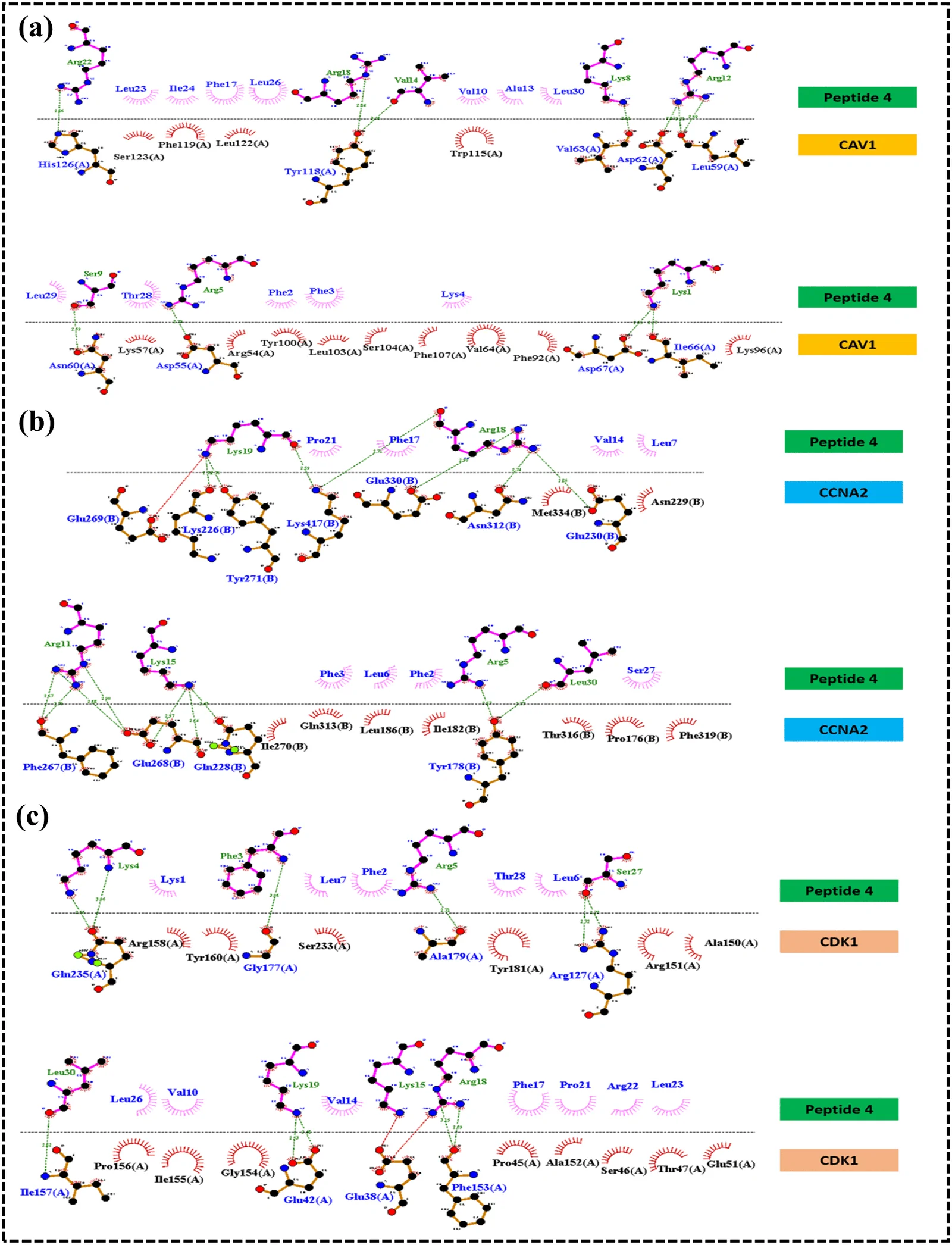
Intermolecular interactions among docked complexes of peptide 4 and host proteins (a) CAV1 (b) CCNA2 (c) CDK1.

**Fig 6:**
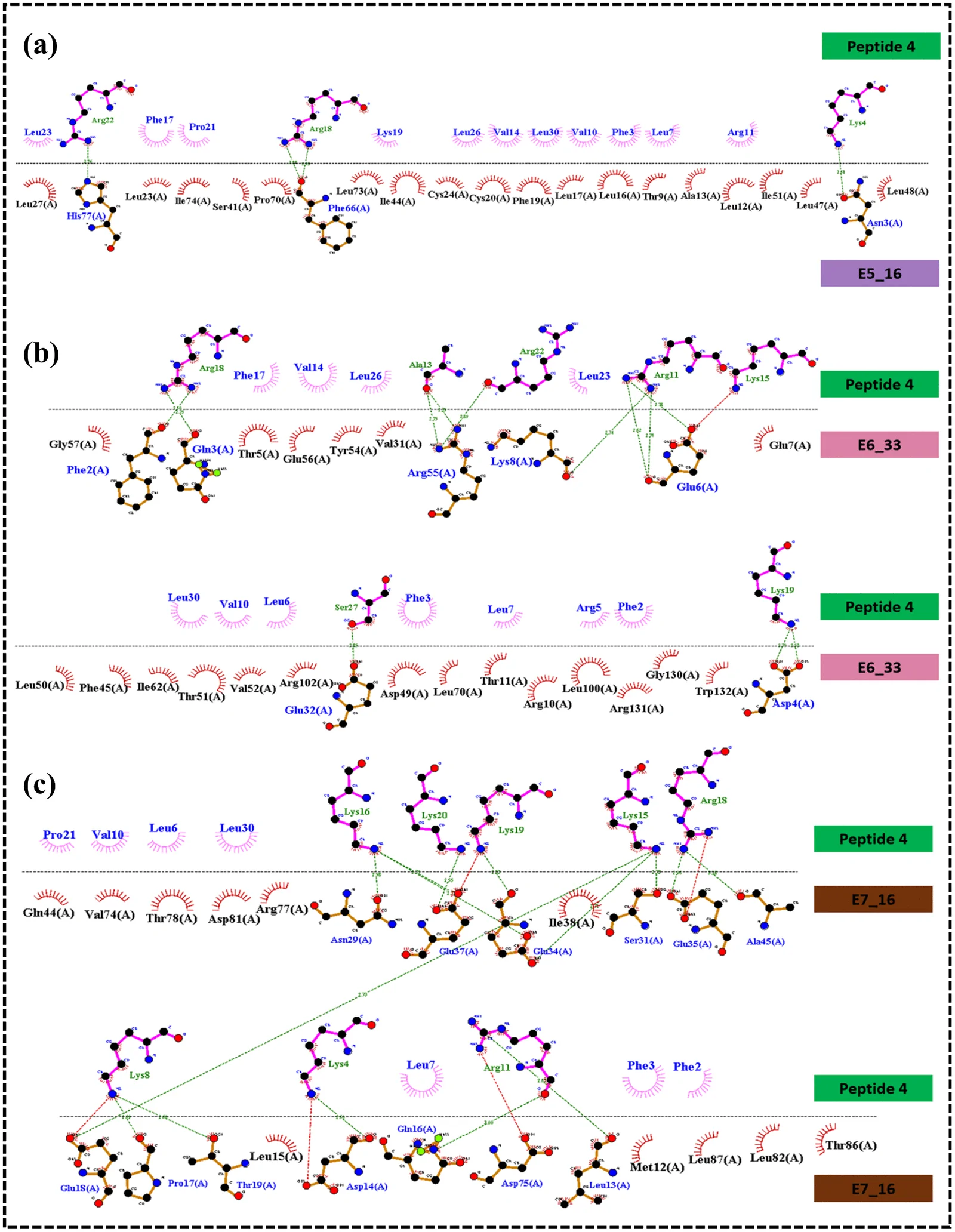
Intermolecular interactions among docked complexes of peptide 4 and HPV proteins (a) E5_HPV16 (b) E6_HPV33 (c) E7_HPV16.

**Fig 7:**
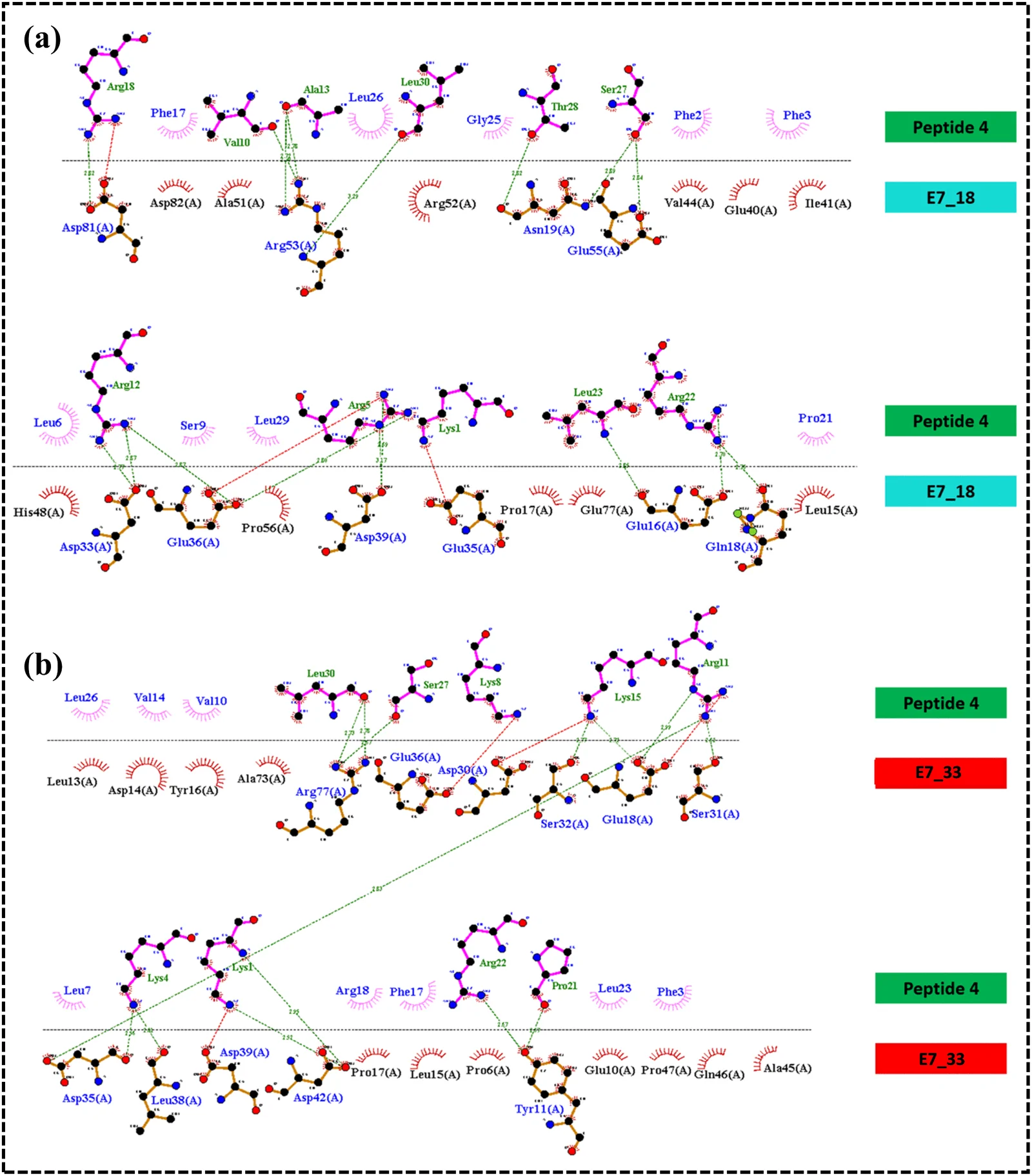
Intermolecular interactions among docked complexes of peptide 4 and HPV proteins (a) E7_HPV18 (b) E7_HPV33.

### Molecular dynamics simulation of docked complexes

The stability and conformational dynamics of the host and HPV protein–peptide (Peptide 4) complexes were analysed using several key molecular dynamics parameters. These analyses provided comprehensive insights into the structural stability and binding behaviour of the complexes during the 100 ns simulation (Figs 8–10). Replicative simulations of all the complexes with different initial velocities were performed to sample the equilibrium state adequately and confirmed the absolute stability (Compared RMSD of all the complexes of the inhibitor, which are presented in the S1 Fig). The molecular dynamics simulation results (Replica 1) are presented in tabulated form in S9 Table and S10 Table.

**Fig 8:**
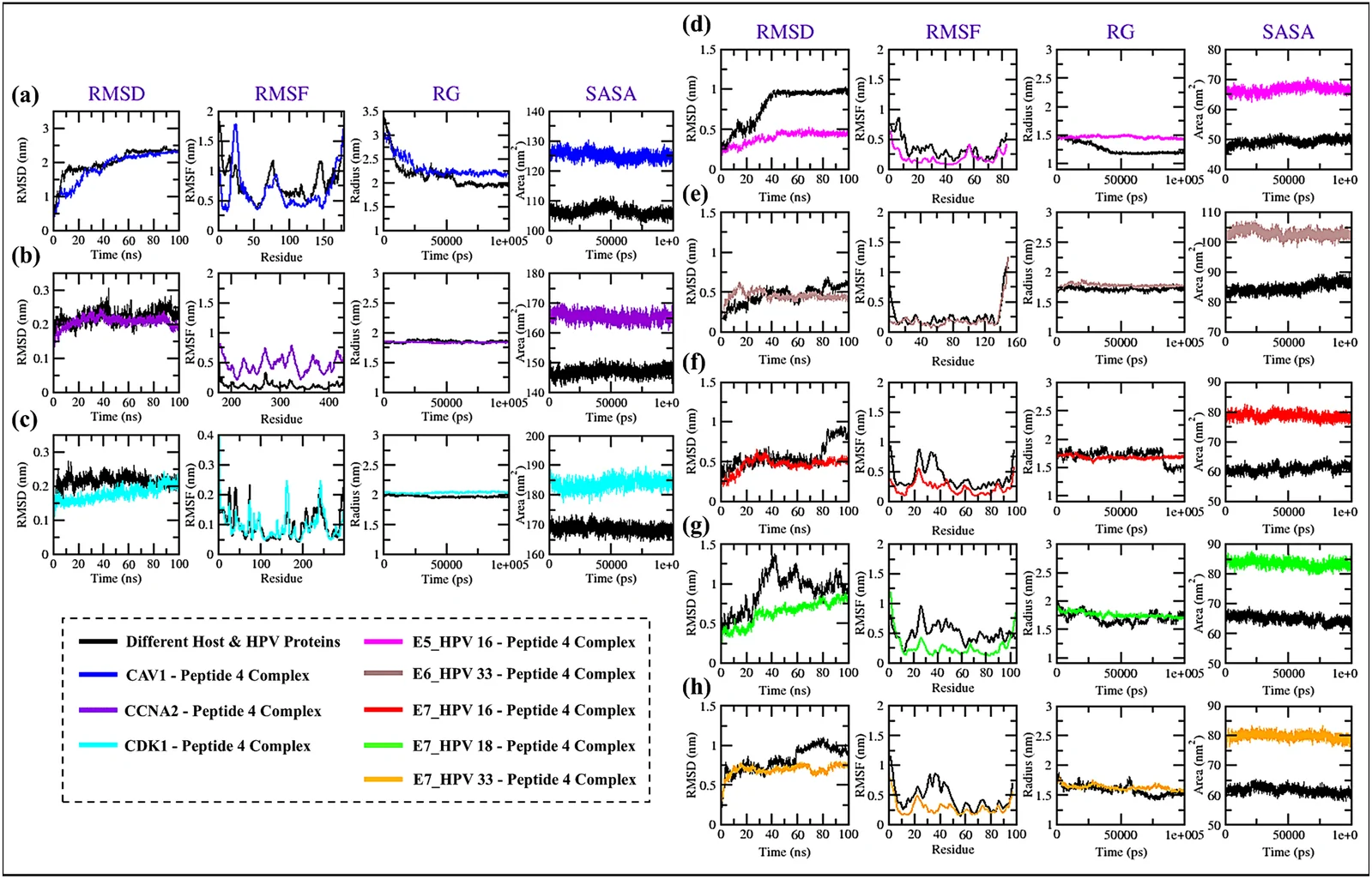
Molecular dynamics simulations (RMSD, RMSF, Rg and SASA) of Host apo-proteins and complexes with peptide 4 in (a) CAV1 (b) CCNA2 (c) CDK1. HPV apo-proteins and complexes with peptide 4 in (d) E5_HPV16 (e) E6_HPV33 (f) E7_HPV16 (g) E7_HPV18 and (h) E7_HPV3.

**Fig 9:**
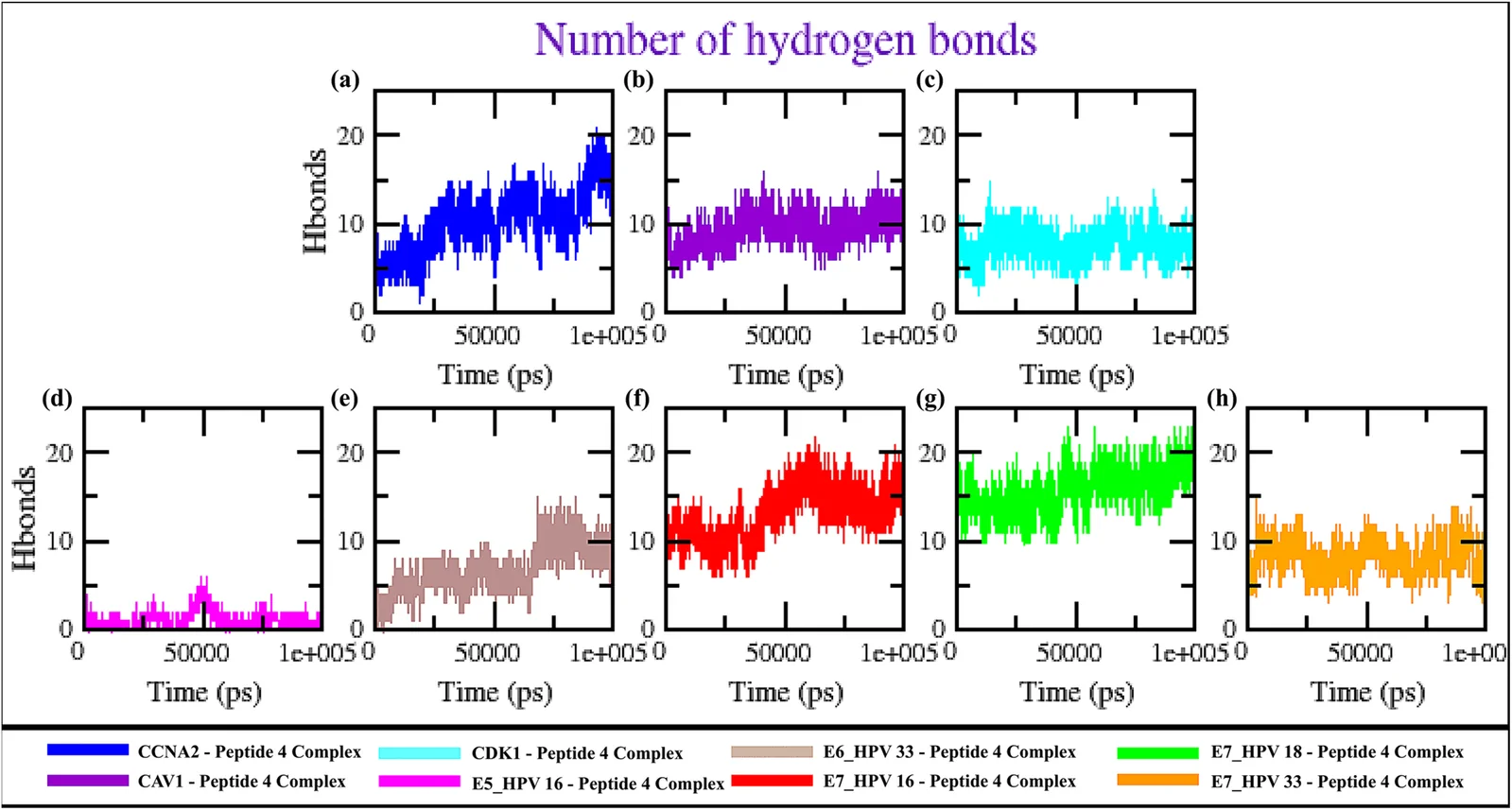
Intermolecular hydrogen bonds of target proteins and peptide 4 complexes (a) CAV1 (b) CCNA2 (c) CDK1 (d) E5_HPV16 (e) E6_HPV33 (f) E7_HPV16 (g) E7_HPV18 and (h) E7_HPV3.

**Fig 10:**
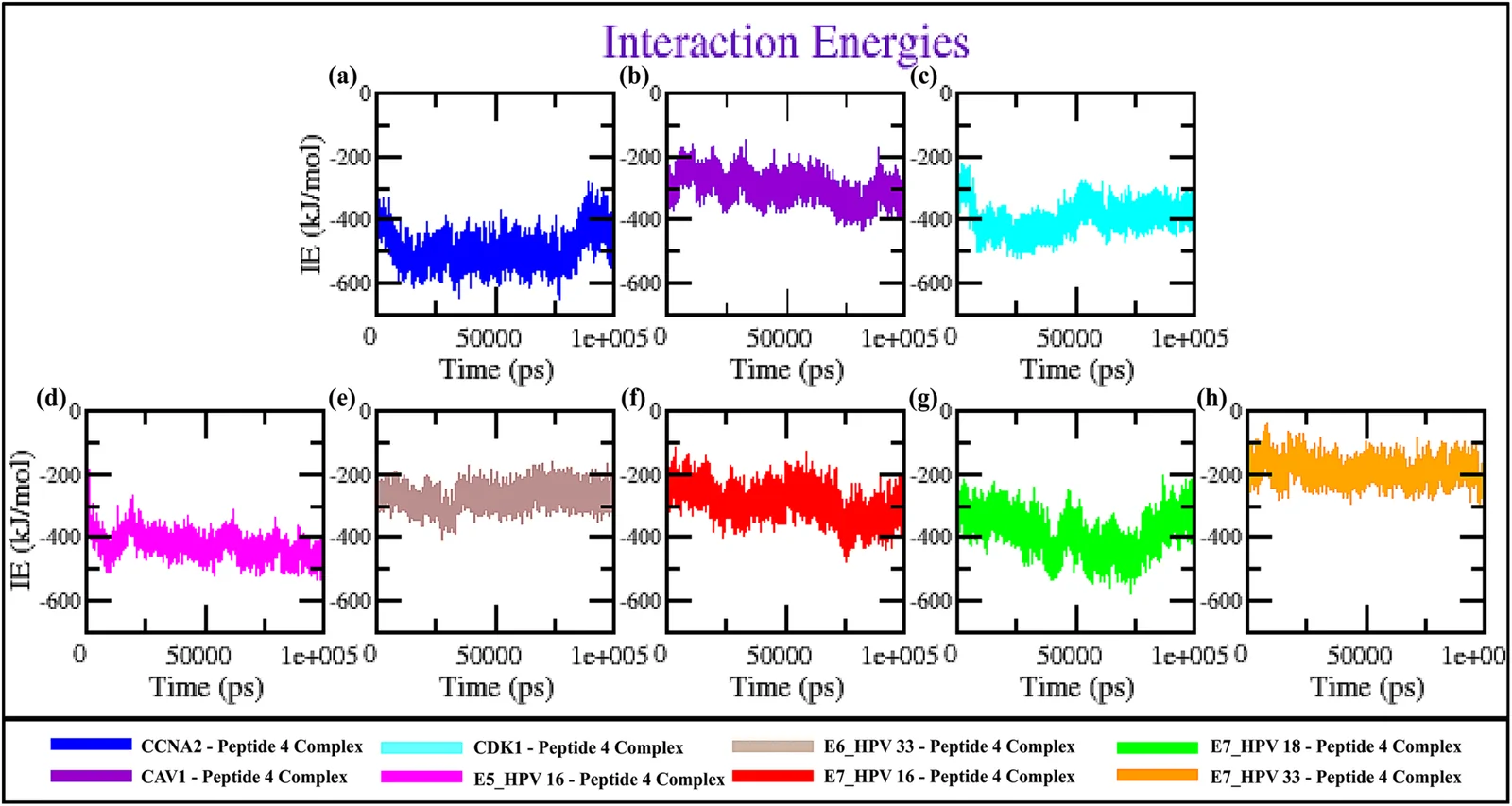
Interaction energies of target proteins and peptide 4 complexes (a) CAV1 (b) CCNA2 (c) CDK1 (d) E5_HPV16 (e) E6_HPV33 (f) E7_HPV16 (g) E7_HPV18 and (h) E7_HPV3.

#### RMSD analysis.

The RMSD was evaluated to assess the structural deviation of both unbound and peptide-bound protein systems over the simulation time. Among host proteins, the average RMSD values were 2.03 ± 0.36 nm (CAV1), 0.22 ± 0.02 nm (CCNA2), and 0.21 ± 0.02 nm (CDK1). For unbound HPV proteins, RMSD values were 0.81 ± 0.2 nm (E5_HPV 16), 0.46 ± 0.1 nm (E6_HPV 33), 0.57 ± 0.1 nm (E7_HPV 16), 0.91 ± 0.2 nm (E7_HPV 18), and 0.82 ± 0.15 nm (E7_HPV 33).

Upon complexation with Peptide 4, the RMSD values for host proteins were 1.87 ± 0.4 nm (CAV1-Peptide 4), 0.21 ± 0.01 nm (CCNA2-Peptide 4), and 0.18 ± 0.02 nm (CDK1-Peptide 4). For HPV protein complexes, RMSD values were 0.4 ± 0.07 nm (E5_HPV 16), 0.45 ± 0.05 nm (E6_HPV 33), 0.47 ± 0.08 nm (E7_HPV 16), 0.63 ± 0.1 nm (E7_HPV 18), and 0.70 ± 0.06 nm (E7_HPV 33) (Fig 8a-h). Based on the comparative analysis of both replicates of all the complexes (S1 Fig), it was shown that with different initial velocities to adequately sample the equilibrium state, which confirmed that the absolute stability had achieved a structural equilibrium during the 100 ns simulation time

#### RMSF analysis.

RMSF was used to quantify the flexibility of individual residues across the simulation trajectory. RMSF values for host proteins and their respective complexes were CAV1 (0.79 ± 0.27 nm) and CAV1–Peptide 4 (0.68 ± 0.33 nm), CCNA2 (0.11 ± 0.05 nm) and CCNA2–Peptide 4 (0.47 ± 0.13 nm), CDK1 (0.10 ± 0.04 nm) and CDK1–Peptide 4 (0.10 ± 0.04 nm).

In the HPV proteins, RMSF values were recorded as E5_HPV 16 (0.34 ± 0.16 nm) and E5_HPV 16–Peptide 4 (0.20 ± 0.10 nm), E6_HPV 33 (0.25 ± 0.18 nm) and E6_HPV 33–Peptide 4 (0.20 ± 0.16 nm), E7_HPV 16 (0.40 ± 0.18 nm) and E7_HPV 16–Peptide 4 (0.23 ± 0.10 nm), E7_HPV 18 (0.50 ± 0.14 nm) and E7_HPV 18–Peptide 4 (0.28 ± 0.18 nm), E7_HPV 33 (0.45 ± 0.20 nm) and E7_HPV 33–Peptide 4 (0.28 ± 0.11 nm) (Fig 8a-h).

#### Rg analysis.

Rg was analyzed to understand the compactness and structural folding of the protein systems. The Rg values for host proteins and their peptide complexes were CAV1 (2.17 ± 0.27 nm) and CAV1–Peptide 4 (2.33 ± 0.30 nm), CCNA2 (1.85 ± 0.01 nm) and CCNA2–Peptide 4 (1.84 ± 0.01 nm), CDK1 (1.97 ± 0.01 nm) and CDK1–Peptide 4 (2.03 ± 0.01 nm).

For HPV proteins and complexes, Rg values were: E5_HPV 16 (1.27 ± 0.10 nm) and E5_HPV 16–Peptide 4 (1.47 ± 0.01 nm), E6_HPV 33 (1.73 ± 0.02 nm) and E6_HPV 33–Peptide 4 (1.79 ± 0.02 nm), E7_HPV 16 (1.70 ± 0.10 nm) and E7_HPV 16–Peptide 4 (1.69 ± 0.02 nm), E7_HPV 18 (1.71 ± 0.08 nm) and E7_HPV 18–Peptide 4 (1.76 ± 0.04 nm), E7_HPV 33 (1.59 ± 0.07 nm) and E7_HPV 33–Peptide 4 (1.63 ± 0.04 nm) (Fig 8a-h).

#### Analysis of SASA.

The SASA values were examined to determine the extent of solvent exposure on the protein surfaces. The SASA values for host proteins and their complexes were CAV1 (106.63 ± 1.35 nm^2^) and CAV1–Peptide 4 (125.4 ± 1.33 nm^2^), CCNA2 (147.19 ± 1.23 nm^2^) and CCNA2–Peptide 4 (165.59 ± 1.37 nm^2^), CDK1 (168.7 ± 1.32 nm^2^) and CDK1–Peptide 4 (183.9 ± 1.62 nm^2^).

In the case of HPV proteins, SASA values were: E5_HPV 16 (49.38 ± 1.00 nm^2^) and E5_HPV 16–Peptide 4 (66.77 ± 1.08 nm^2^), E6_HPV 33 (84.98 ± 1.50 nm^2^) and E6_HPV 33–Peptide 4 (103.13 ± 1.23 nm^2^), E7_HPV 16 (61.12 ± 1.11 nm^2^) and E7_HPV 16–Peptide 4 (78.9 ± 1.01 nm^2^), E7_HPV 18 (65.03 ± 1.17 nm^2^) and E7_HPV 18–Peptide 4 (83.64 ± 1.15 nm²), E7_HPV 33 (61.71 ± 1.09 nm²) and E7_HPV 33–Peptide 4 (79.97 ± 0.99 nm^2^) (Fig 8a-h).

#### Intermolecular hydrogen bonding.

The number and persistence of intermolecular hydrogen bonds (H-bonds) were used to gauge the interaction strength and stability of the protein–peptide complexes. The CAV1–Peptide 4 complex formed up to 21 H-bonds, with 11 consistently maintained throughout the 100 ns simulation. CCNA2–peptide 4 and CDK1–peptide 4 complexes exhibited 16 and 15 H-bonds, of which 10 and 8, respectively, were persistent.

For HPV complexes, frequent H-bonds were observed: one out of six (E5_HPV 16–Peptide 4), six out of 15 (E6_HPV 33–Peptide 4), 14 out of 22 (E7_HPV 16–Peptide 4), 17 out of 23 (E7_HPV 18–Peptide 4), and eight out of 15 (E7_HPV 33–Peptide 4) (Fig 9a-h).

#### Interaction energy assessment.

Binding interaction energies, calculated as the Lennard-Jones short-range potential, revealed the strength of protein–peptide interactions. Host protein complexes showed binding energies of –490.09 kJ/mol (CAV1–Peptide 4), –296.04 kJ/mol (CCNA2–Peptide 4), and –392.15 kJ/mol (CDK1–Peptide 4).

HPV protein–peptide complexes displayed interaction energies of –421.35 kJ/mol (E5_HPV 16), –271.43 kJ/mol (E6_HPV 33), –289.94 kJ/mol (E7_HPV 16), –383.13 kJ/mol (E7_HPV 18), and –185.24 kJ/mol (E7_HPV 33) (Fig 10a-h).

### Validation of docked complexes using MM-GBSA energy profiling

The MM-GBSA approach was employed to evaluate the binding free energies and thermodynamic stability of the docked host and HPV protein-peptide 4 complexes. MM-GBSA binding free energies assessed interactions’ favorability following the MD simulation and stabilization of the complexes. Table 5 summarizes the calculated binding free energies for all studied protein-peptide complexes. Among the host proteins, the binding free energies were recorded as −139.97 kcal/mol for CAV1-Peptide 4, −92.31 kcal/mol for CCNA2-Peptide 4, and −81.71 kcal/mol for CDK1-Peptide 4 complexes. These values indicate favorable and stable binding, particularly for the CAV1-peptide 4 complex. Whereas for HPV protein-peptide complexes such as E5_HPV 16 -Peptide 4, E6_HPV33-Peptide 4, E7_HPV16 -Peptide 4, E7_HPV18 -Peptide 4 and E7_HPV33 -Peptide 4 yielded binding energies of −77.62 kcal/mol, −41.02 kcal/mol, −136.7 kcal/mol, −148.05 kcal/mol and −81.89 kcal/mol, respectively. Among the HPV complexes, E7_HPV18-Peptide 4 and E7_HPV16-Peptide 4 exhibited the most favorable binding profiles. Fig 11a-b illustrates the distribution of MM/GBSA energy components across all complexes, including van der Waals (VDWAALS), electrostatic energy (EEL), polar solvation (EGB), and non-polar solvation (ESURF) energy contributions.

**Table 5:** MM-GBSA method Calculated Binding Energies for Host and HPV Protein-Peptide Complexes.

Complex	ΔVDWAALS*	ΔEEL*	ΔEGB*	ΔESURF*	ΔGGAS*	ΔGSOLV*	ΔTOTAL*
CAV1-Peptide 4	−136.07	−2378.36	2397.45	−22.99	−2514.43	2374.46	−139.97
CCNA2-Peptide 4	−77.48	−1472.74	1470.7	−12.8	−1550.21	1457.9	−92.31
CDK1-Peptide 4	−98.29	−375.94	407.22	−14.7	−474.23	392.52	−81.71
E5_HPV16-Peptide 4	−114.09	217.93	−166.66	−14.8	103.84	−181.45	−77.62
E6_HPV33-Peptide 4	−60.44	586.37	−557.09	−9.86	525.93	−566.95	−41.02
E7_HPV16-Peptide 4	−67.96	−3664.16	3609.72	−14.3	−3732.12	3595.42	−136.7
E7_HPV18-Peptide 4	−83.14	−2359.63	2310.37	−15.65	−2442.77	2294.71	−148.05
E7_HPV33-Peptide 4	−47.04	−2421.58	2396.08	−9.36	−2468.62	2386.73	−81.89

*All units are in kcal/mol.

**Fig 11:**
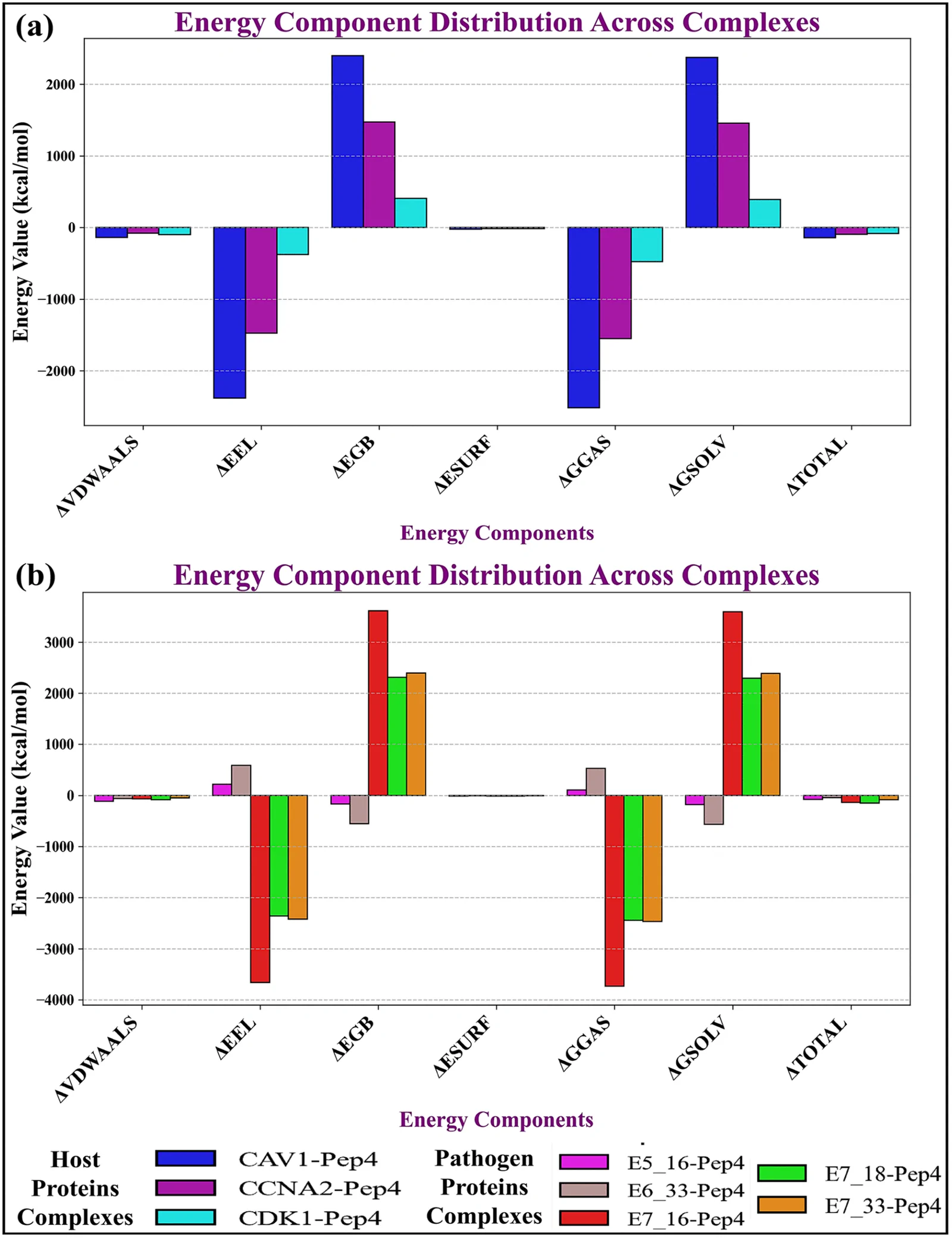
MMGBSA binding-free energy contribution of various interactions between the target proteins and peptide 4 (a) Host proteins (CAV1, CCNA2 and CDK1)-peptide 4 complexes (b) HPV proteins (E5_HPV16, E6_HPV33, E7_HPV16, E7_HPV18 and E7_HPV33)-peptide 4 complexes.

### Decomposition analysis based on MM-GBSA energy profiling

To further strengthen the energetic claims derived from the MM-GBSA binding free energy calculations, per-residue energy decomposition analysis was performed for all eight target protein-Peptide 4 complexes across the 100 ns trajectories presented in Fig 12a-h. Per-residue decomposition analysis provided a detailed residue-level contribution to the total binding free energy at each specific host or viral protein binding interface. This enabled the identification of the residues contributing most significantly to the overall stability of each complex. The major energetically contributing receptor residues to the CAV1-Peptide 4 interaction (Fig 12a) were found to be Asp55, Leu59, Asp61, Asp62, Val63, Val64, Asp70, and Trp115. Correspondingly, the major energetically contributing ligand residues were found to be Phe2, Phe3, Arg5, Leu6, Arg12, Phe17, Arg18, Pro21, Ile24, Leu26, Thr28, and Leu29. In contrast, for the CCNA2-Peptide 4 interaction (Fig 12b), major receptor contributions to binding were observed for residues Tyr178, Asp181, Ile182, Glu230, Glu268 and Glu269. Conversely, the major energetically contributing ligand residues were found to be Phe2, Phe3, Leu6, Arg11, Lys15, Arg18, Pro21 and Leu23. For the CDK1-Peptide 4 interaction (Fig 12c), significant receptor contributions to binding were observed for residues Glu38, Glu42, Pro45, Thr47, Arg127, Glu154, Pro156, Tyr181 and Gln235, while significant ligand contributions to binding were observed for residues Phe2, Phe3, Lys15, Phe17, Arg18, Lys19, Lys20, Pro21, Leu26 and Leu30. The majority of HPV viral proteins had the lowest negative binding free energies when compared to the host proteins. As such, they represented the best candidates for a structural basis of inhibition studies. Among these proteins, the E5_HPV16-Peptide 4 complex (Fig 12d) represented the numbers of receptor residues Leu16, Phe19, Cys20, Leu23, Cys24, Leu27, Leu33, Ile44, and Ile74 which contributed to binding. Similarly, several residues Phe3, Leu6, Leu7, Val10,Val14, Phe17, Pro21, Leu26 and Leu29 also made significant contributions on the ligand side. For the E6_HPV33-Peptide 4 complex (Fig 12e), significant receptor contributions to binding were observed for residues Asp4, Glu6, Asp49, Val53, Tyr54, Arg77, and His78, where significant ligand contributions to binding were observed for residues Phe2, Phe3, Val10, Val14, Lys15, Ile24, Leu26, and Leu30. For the E7_HPV16-Peptide 4 complex (Fig 12f), receptor residues Leu13, Glu16, Glu18, Glu33, Glu34, Glu37, Ile38, Val74, and Asp75 dominated the energetic landscape, while significant ligand contributions to binding were observed for residues Phe3, Arg5, Leu7, Lys8, Arg11, Arg12, Lys15, Lys16, Arg18, Lys19, and Leu26. The E7_HPV18-Peptide 4 complex (Fig 12g) displayed substantial receptor contributions to binding for residues Leu15, Glu16, Glu20, Asp33, Glu35, Glu36, Asp39, and Asp81 with Lys1, Phe3, Arg5, Leu6, Arg12, Arg18, Pro21, Arg22, Leu23, and Ile24 of Peptide 4 as the dominant residues. Finally, for the E7_HPV33-Peptide 4 complex (Fig 12h), significant receptor contributions to binding were observed for receptor residues Leu15, Glu18, Asp30, Asp35, Glu36, Asp39, and Asp42, while ligand contributions to binding were observed for residues Lys4, Arg5, Lys8, Val10, Arg11, Arg12, Val14, Lys15, Arg18, and Leu23. Overall across all complexes, decomposition analysis showed that the arginine (Arg5, Arg11, Arg12, Arg18) and hydrophobic (Phe2, Phe3, Leu6, Leu7, Leu26) residues of Peptide 4 were the principal energetically contributing residues. This pattern is consistent with the cationic and amphipathic character of the peptide, driving favourable electrostatic and van der Waals interactions with complementary acidic and hydrophobic receptor residues across the diverse host and viral protein binding interfaces.

**Fig 12:**
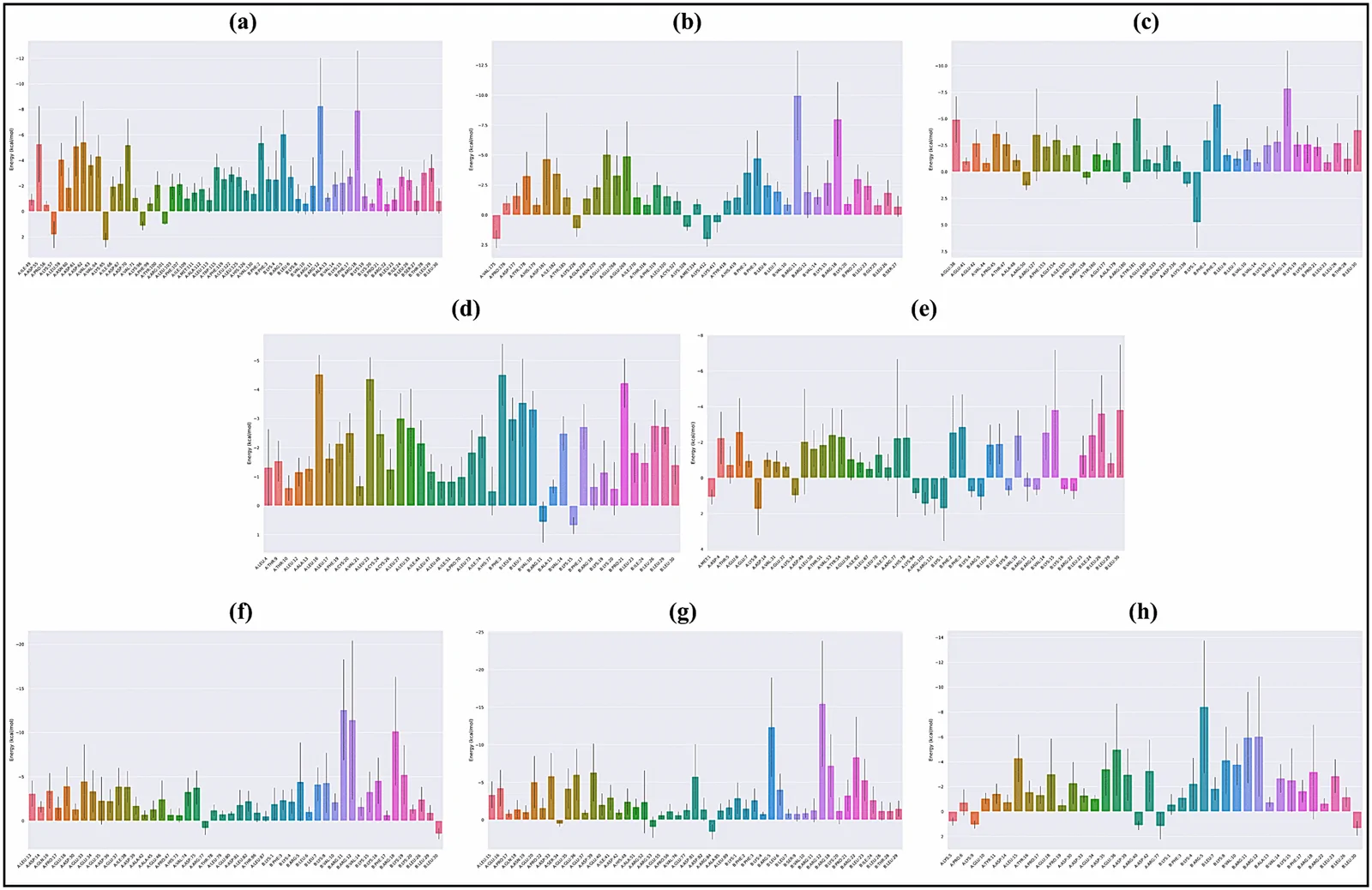
Decomposition analysis of all complexes after MMGBSA-based binding free energy calculation (a) CAV1-Peptide 4, (b) CCNA2-Peptide 4, (c) CDK1-Peptide 4, (d) E5_HPV16-Peptide 4, (e) E6_HPV33-Peptide 4, (f) E7_HPV16-Peptide 4, (g) E7_HPV18 -Peptide 4 and (h) E7_HPV33)-peptide 4 complexes.

## Discussion

Viral carcinogenesis is responsible for genomic instability, DNA damage, aberrations and mutational load. HPV infection is a leading risk factor for head and neck cancers and is being increasingly reported worldwide. An efficient therapeutic regimen against HPV-led HNSCC can be to target consequently the viral and host target proteins that play crucial roles for the infection led tumorigenesis. The present study identified major HR-HPV subtypes that are closely associated with the host proteins (CDK1 and CCNA2) responsible for the cell cycle machinery. The dual functional antiviral and anticancer peptides screened were highlighted to inhibit the target proteins to establish a potential antiviral and anticancer therapeutic regimen for HPV-associated HNSCC. A similar approach was employed by Mansouri and team, where they identified novel furo-[2,3-d]pyrimidine–1,3,4-oxadiazole hybrid derivatives as antiviral agents against mutant varicella-zooster virus and anticancer agents against fibrosarcoma, breast and lung cancer cell lines [64].

Topological metrics assessed the interactome nodes and highlighted the density of the network, and were majorly contributed by the central points designated as EGFR, BRCA1, CDK1, CAV1, TERT, and CCNA2 among the host proteins. The viral central points were identified to be the E5 and E7 proteins of HPV16, E7 and E6 proteins of HPV33, and E7 protein of HPV 18. Among the host targets, EGFR, TERT, and CCNA2 were enriched in the HPV signalling pathway, whereas CDK1 and CCNA2 were enriched in the viral carcinogenesis pathway. All identified host targets were directly involved in tumor progression signalling. Further, clinical survival relevancy depicted EGFR, CDK1, CCNA2 and CAV1 to be more critical to HNSCC progression. Mutational burden showed EGFR to be highly mutated when compared to CDK1, CCNA2, and CAV1, which were thereafter considered potential therapeutic targets for HNSCC. The roles of CCNA2 and CDK1 were validated by the expression patterns observed in HPV-positive samples.

The host protein target CDK1 regulates the transition through different phases of the cell division cycle and activates transcription factors. CDK1 essentially promotes epithelial-mesenchymal transition (EMT) by regulating ∆Np63α, which is in turn involved in cell adhesion, migration and motility [65]. The tumorigenic role of CCNA2 was recorded by the decreased cell proliferation in oral squamous cancer by the suppression of CCNA2 on silencing lincRNA AFAP-AS1 [66]. Moreover, the direct involvement of CCNA2 in cell proliferation was evidenced by the enrichment of pathways in cancer (hsa05200). microRNA miR-381-3p on inhibiting CCNA2 and MET has shown drastic reduction in the cell cycle and EMT progression [67]. Caveolin-1, encoded by the CAV1 gene, is a critical membrane protein that is essential for cell signaling and transport. It is expressed in various cells including cancer cells and have been found to be up-regulated in multiple cancers. CAV1 is reportedly involved in cell proliferation, invasion, death as well as metabolism of lipids [68]. A recent report also suggested the role of CAV1 in inhibiting ferroptosis thereby leading to progressive HNSCC [69]. The major biological processes of CAV1 include the regulation of cell migration, cell communication, signaling, tissue remodelling, and fibroblast proliferation. Therefore, overexpressed CAV1 is a key factor in tumor migration and proliferation.

The viral E5 and E7 proteins of HPV 16 are considered as oncogenic proteins with effective roles in virus led tumor proliferation. The gene ontology terms enriched for E5 protein include mitotic cell cycle and cell adhesion, indicating its role in these processes [70]. E5 promotes immune evasion by altering the pH and subsequent acidification of the organelles. E6 oncoprotein of HPV 33 has been reported to inhibit tumor suppressor proteins, resulting in an immunosuppressive environment [71]. The involvement of E7 oncoprotein in malignancy can be attributed to the binding of E7 protein to retinoblastoma 1 protein (Rb1) and modulating its function [72,73].

The host-pathogen interactome analysis identified crucial interlinks among the host and viral target proteins suggesting the identified hub-genes to be critical for therapeutic targeting. For instance, the E5 protein interacting with CAV1 and EGFR are significant interactions as E5 protein on interaction with EGFR activates the receptor and the subsequent signaling cascading initiating the activation of several proto-oncogenes [74]. The interaction with CAV1 leads to the redistribution of the CAV1 protein. CAV1 acts as a co-stimulatory signal for TCR, which further results in T-cell proliferation and activation of NF-κB signaling. The redistribution of CAV1 aids the virus in invading the host cells via caveolae-mediated endocytosis. Once the virus undergoes internalization, it activates p38 MAPK and Src, which is followed by CAV1 and Rac1 activation [75]. HPV infection through clathrin-mediated endocytosis is also reported to be dependent on CAV1, suggesting the importance of the direct association of HPV and CAV1 protein. In addition, EGFR is a component of the Ras-p42/44 MAPK signaling is compartmentalized due to CAV1 [76]. From the interactome, it is evident that the E7 oncoproteins of HPV 16, 18, and 33 interact with CDK1, CCNA2, and BRCA1, respectively. This can be supported by the report of CDK1 being activated by E7 protein. Modulation of the CDKs/cyclins in turn results in the impairment of G2 checkpoint leading to chromosomal instability [77]. Activated CDK1 regulates cell cycle aiding tumor cell proliferation [78]. Moreover, E6/E7 present keratinocytes were found to have over-expressed genes responsible for the promotion of G2-M phase transition [79]. CCNA2 also activates CDK2 regulating G1-S and G2-M phase transition [80]. The E7 protein inhibits the Rb protein, leading to the release of E2F protein and subsequent binding to CCNA2 promoter region. This binding leads to the constitutive cell cycle activation and promotion of G1 to S phase [81,82]. E6 protein degrades p53 contributing to the evasion of apoptosis. E6 interaction with BRCA1 suggests the role of E6 to suppress the activity of BRCA1 inhibition of Myc E-box mediated transactivation.

The established interactome hub-genes were then validated for its potential as therapeutic targets. The over-expressed, critically significant median overall survival and mutational landscape has filtered the CDK1, CCNA2 and CAV1 as potential host targets for further analyses. The non-toxic, cell penetrating, anti-inflammatory, non-hemolytic antiviral peptides with anticancer properties were screened and modelled to dock with the viral and host targets. Among the four peptides, peptide 4, also known as Hydrophis cyanocinctus cathelicidin (Hc-CATH) or sea snake cathelicidin, showed a comparatively high average binding affinity with all the targets, suggesting its effect as an antiviral and anticancer therapeutic. It is a naturally occurring antimicrobial peptide originally isolated from the venom gland of the sea snake *Hydrophis cyanocinctus*, deposited in the Antimicrobial Peptide Database (APD6) under accession ID AP02569.The anti-viral activity of Peptide 4 (AP02569 or Hc-CATH) against RNA viruses causing Zika virus (ZIKV) has been previously reported, and its anti-bacterial, anti-fungal, anti-inflammatory, and anti-sepsis properties have also been documented [83]. The binding of the peptide to the active site residue of CDK1 (R127-IPR008271) indicates the ability of the peptide to interfere with the catalytic activity of the protein. In case of CAV1, protein-peptide complex showed several interactions near the conserved site spanning from 68 to 75 amino acid residues (IPR001612) and hydrophobic interactions at F92 and K96 spanning the region interacting with CAVIN3 (IPR001612), also confirming the inhibitory effect of the peptide. The cyclin domain (IPR048258) spanning from 211–242 amino acid residues specifically confers substrate specificity to CCNA2. The peptide showed two H-bond interactions (K226 and Q228) and one hydrophobic interaction (N229) with respect to the cyclin domain. The functional domain of E5 protein of HPV 16 spans across 11–82 amino acid residue position (IPR004270), while for E6 protein of HPV 33, it is around 5–142 amino acid residue position (IPR001334) and for E7 protein, the functional domain, IPR000148 is common across all three subtypes. The various canonical and non-canonical interactions with the viral protein domain indicated the inhibitory effect of the peptide.

MD simulations provide information about the dynamics of complexes between the host and the HPV protein-peptide 4. RMSD trajectories (in both replicate runs) showed that the complexes have achieved a structural equilibrium during the 100 ns simulation time, with a minimum deviation from the starting conformations; this indicates that they have a low structural instability, as presented in Fig 8 and S1 Fig, consistent with prior MD simulation studies that have demonstrated the reliability of 100 ns trajectories in capturing the structural dynamics and interaction profiles of protein complexes [84–86]. Therefore, the results indicate that the complexes are structurally stable under physiological conditions, and they confirm the reliability of the docked complexes. Further, Rg analysis showed that the host and viral protein-peptide complexes are compact. The Rg values obtained for the same type of complexes were generally constant along the trajectory, and the compactness was reflected by the structure of the proteins after binding the peptides. In addition, the SASA of the complexes shows a stable solvation layer surrounding them, as well as a sufficient shielding of hydrophobic regions. The SASA values suggest a good solvation of the proteins, which is essential for the structural stability of proteins in an aqueous environment. Finally, the SASA values also showed that the binding of the peptides produces changes in the surface-exposed hydrophobic residues, confirming that the binding of the peptides modifies the solvation characteristics and the surface topology of the proteins. The observed hydropathy behaviour confirms the structural stability of the complexes. Additionally, the intermolecular hydrogen bonding analysis provided additional evidence on the stability of the complexes. A significant proportion of the complexes analysed formed a large number of stable and persistent hydrogen bonds during the entire duration of the simulation. It is reasonable to infer that these hydrogen bonds contributed to the thermal stability and specificity of the complexes. Furthermore, the correlation between the hydrogen bonding frequencies and SASA values supports the hypothesis that there exists a synergy between the contributions of hydrogen bonds and solvation in maintaining the stability of the complex conformations as it. Furthermore, the binding free energies calculated using the MM-GBSA method supported the structural stability observed in MD simulations and confirmed the high affinity of peptide 4 for multiple host and HPV target proteins. Specifically, the most favorable interactions were detected in the CAV1 and E7_HPV18 complexes, and these interactions may be associated with a higher biological activity. The energy component decomposition demonstrated that the van der Waals and electrostatic contributions play a major role in defining the interaction energies.

Collectively, the results of molecular docking, MDS, and MM-GBSA studies demonstrated that peptide 4 is a stable and high affinity binder for multiple host and HPV protein targets. These findings demonstrated that it has the potential to act as a therapeutic agent with dual antiviral and anticancer activities. Furthermore, given its ability to bind to key host-pathogen interaction sites involved in HPV-associated HNCs, it can be used as a valuable scaffold for further optimization and development as a multifunctional therapeutic agent. In addition, the treatment with peptide 4 could potentially prevent the activation of oncogenes and thus prevent further worsening of tumorigenesis. This study’s multi-server, multi-descriptor approach to evaluating Peptide 4 by combining descriptors for charge, hydrophobicity, toxicity, stability, CPP prediction, hemolysis, antigenicity, inflammation inhibition, and cancer cell killing together embodies this general concept. In fact, it follows the well-established tradition in the literature that effective choice of molecular descriptors and thoroughness of computational protocols are essential to predictability of bioactivity and PK stability for small molecules being considered as new drugs and thus represents the pharmacological translation capability of Peptide 4 as a scaffold for treatment of HNSCC caused by HPV [87]. It is also vital to acknowledge that this study is an entirely computational endeavour, therefore these results represent hypothetical expectations that have to be empirically validated prior to drawing any clinical conclusion from it. Therefore, validating the theoretically predicted anti-cancer activity of Peptide 4 through cytotoxicity profiling using the cell viability assay MTT, would allow for the establishment of its half-maximal inhibitory concentration (IC₅₀), and selectivity index towards malignant epithelial cells relative to normal epithelial cells -an approach that has been successfully employed in analogous multitargeted computation studies followed by MMT-based experimental confirmation [88]. This will ultimately determine if the theoretically expected anti-cancer activity of Peptide 4 is reflected in an actual inhibition of proliferation of malignant epithelial cells under in vitro conditions. The validation process described here has been standard practice in the field of peptide therapeutics; wherein computational prediction of ligand interaction is typically followed by experimental verification of binding affinity, and subsequently, demonstration of cytotoxic effects on cancerous vs. noncancerous cells, prior to investigating their efficacy in more complex model systems [89].

## Conclusion

Human papillomavirus infects various head and neck regions, leading to uncontrolled tumor growth. The viral oncoproteins activate the signaling mechanisms aiding in progressive tumorigenesis. The host-pathogen interactome identified key hub-nodes interacting among the viral proteins and host proteins. CDK1, CCNA2, EGFR, CAV1, BRCA1 and TERT showed close interactions with E5, E6 and E7 proteins of HPV subtypes. Physicochemical property screening revealed antiviral-anticancer dual-functional peptides of peptide 4 (AP02569), which showed a high binding affinity for viral and host protein targets. The comprehensive MD simulations demonstrated that peptide 4 (AP02569) forms stable and energetically favorable complexes with both the host and HPV proteins implicated in HPV-associated pathogenesis. The peptide maintained structural integrity across all complexes, as evidenced by stable RMSD, Rg, SASA, and hydrogen bonding profiles. The minimal fluctuations and overall stable docked complexes of peptide 4 (AP02569) and protein targets validated the stability of the protein-peptide complexes throughout the studied timeframe in the simulation. Notably, MM-GBSA binding free energy calculations further confirmed the strong interaction affinities. This also indicated the use of peptide 4 as a potent therapeutic intervention against HPV-led HNSCC tumours. The clinical translation of peptide-based therapeutics holds significant promise for circumventing virus-induced carcinogenesis, however, the present study findings, being entirely *in silico*, necessitate further experimental validation.

## Supporting information

S1 Table FEA of HNSC over-expressed genes. (DOCX)

S2 Table Pathogen proteins involved in HNSCC. (DOCX)

S3 Table Host proteins involved in HNSCC. (DOCX)

S4 Table Host protein structure with Ramachandran favoured regions. (DOCX)

S5 Table Viral protein structure with Ramachandran favoured regions. (DOCX)

S6 Table Retrieved AVPs, step-by-step screening of all the peptides. (XLSX)

S7 Table Secondary structure analyses of the screened peptides and Controls. (DOCX)

S8 Table Docking validation of Lead Peptide 4 and two controls against all the target proteins through HDOCK. (DOCX)

S9 Table Molecular dynamics simulation analysis on 3 different Host apo-proteins and their complex with Peptide 4. (DOCX)

S10 Table Molecular dynamics simulation analysis on 5 different HPV (Pathogen) apo-proteins and their complex with Peptide 4. (DOCX)

S1 Fig RMSD comparison of all complexes through 100 ns MD Simulation replicate run. (DOCX)
