# Supplementary material for: A dual-functional antiviral and anticancer peptide to target intricate host-pathogen interacting proteins of human papillomavirus associated with head and neck cancer
Source: PLoS One. 2026 Oct 9;21(10):e0359513. doi: 10.1371/journal.pone.0359513 (PMC13652162; doi:10.1371/journal.pone.0359513)
Supplement: Supplementary file 1 [file pone.0359513.s001.docx]

**S1 Table** **FEA of HNSC over-expressed genes**

| **Pathway** | **Fold Enrichment** | **Enrichment FDR** | **No: Genes** | **Genes** |
| --- | --- | --- | --- | --- |
| Pathways in cancer | 1.727 | 1.72E-05 | 82 | ITGA3 ITGA2B RAD51 LAMA3 TRAF1 LAMC2 PTGS2 GLI2 FGF4 IL12RB2 BIRC5 DLL3 LAMB1 ITGA6 IL12RB1 MMP9 E2F1 LAMA1 CCNE1 TGFB1 CDK6 MET WNT2 FGF8 GLI1 IFNG PDGFRB FN1 STAT1 WNT6 SLC2A1 PGF CKS2 PRKCG GNGT1 RAC2 EPO CCNA1 WNT2B IL2RA COL4A2 WNT10A STAT4 FGF5 BRCA2 CCNA2 GNA12 EGFR CDKN2A VEGFC WNT7A FGF17 FGFR4 PTGER1 GNG3 FGF19 HEYL TERT HEY1 FZD6 GNG8 FADD IL7R CXCL8 RAC3 STAT2 PIK3CD CKS1B CCNE2 F2 FZD2 F2R FGF3 COL4A1 WNT7B COL4A5 MMP1 LAMB3 PDGFA COL4A6 IFNA1 NTRK1 |
| Neuroactive ligand-receptor interaction | 2.392 | 5.89E-11 | 75 | NPFFR2 NTSR1 CHRNA4 OXT GPR50 BRS3 GABRE MLNR LHB GRIN2D PPY GABRR2 OPRM1 GABRG2 KISS1R CHRNB4 GHRH IAPP GHSR GRM4 GRIA3 PYY NMUR2 NTS GRP HCRTR2 PTH2R UCN2 GLRA3 GZMA HTR2C CHRNB3 CHRNA6 HTR7 MCHR2 CYSLTR2 GIP PTGER1 UCN F2RL2 TAC3 DRD5 CHRNA5 ADRA1B KISS1 FPR2 APLN RXFP1 CHRNA9 TRHR GPR156 HTR1F HTR1D GRM8 F2 SSTR2 S1PR5 F2R GABRG3 NPW CHRM5 GABRA5 LYPD6 FPR3 GABRD ADRB3 CNR2 PATE2 NMB NPY4R CSH2 MC1R GABRQ SSTR3 NPBWR1 |
| Human papillomavirus infection | 2.226 | 2.07E-08 | 66 | ITGA3 ITGA2B IBSP TNC LAMA3 EIF2AK2 LAMC2 CREB3L3 HES2 PTGS2 LAMB1 ITGA6 E2F1 LAMA1 CCNE1 CDK6 WNT2 COL1A1 PDGFRB ITGB6 FN1 STAT1 WNT6 SPP1 RBPJL ITGB4 CCNA1 WNT2B COL4A2 OASL WNT10A ITGA11 COL2A1 COL6A1 COL6A2 CCNA2 EGFR WNT7A PPP2R2B MX1 ITGA5 COL6A3 HEYL TERT HEY1 COL1A2 FZD6 FADD STAT2 PIK3CD IFNB1 CCNE2 HES7 FZD2 TBPL2 MX2 THBS2 COL4A1 ISG15 WNT7B COL4A5 HES4 LAMB3 COL4A6 IFNA1 ITGA1 |
| PI3K-Akt signaling pathway | 1.955 | 6.14E-06 | 62 | ITGA3 ITGA2B BRCA1 IBSP TNC LAMA3 LAMC2 CREB3L3 FGF4 LAMB1 ITGA6 ANGPT2 OSM LAMA1 CCNE1 CDK6 MET FGF8 COL1A1 PDGFRB ITGB6 FN1 SPP1 PGF GNGT1 EPO ITGB4 NGF IL2RA COL4A2 ITGA11 FGF5 COL2A1 COL6A1 COL6A2 EGFR VEGFC PPP2R2B FGF17 FGFR4 ITGA5 GNG3 COL6A3 COL1A2 GNG8 IL7R PIK3CD IFNB1 CCNE2 CD19 F2R THBS2 FGF3 COL4A1 COL4A5 LAMB3 PDGFA COL4A6 IFNA1 NTRK1 CSH2 ITGA1 |
| Cytokine-cytokine receptor interaction | 2.278 | 4.36E-08 | 60 | TNFRSF12A CCL26 TNFRSF9 RELT IL12RB2 IL11 IL12RB1 OSM IL21R AMH TGFB1 CCL7 IFNG IL1A CCL20 TNFSF4 TNFSF18 TNFSF11 TNFRSF8 INHBA IL13RA2 ACVR1C INHA IL1B CD70 EPO CCL25 NGF IL2RA IL21 CXCL9 CD27 XCL1 CXCL14 IFNK CXCL13 GDF6 IL24 BMP10 CSF2 IL31RA IL7R CXCL10 CXCL11 CXCL8 IFNB1 CCL11 CCR8 IFNL1 BMP8A IFNL2 IFNE TNFRSF4 TNFRSF18 IFNL3 IL27 IFNA1 IL31 CSH2 TNFRSF25 |
| Neutrophil extracellular trap formation | 3.290 | 6.93E-14 | 56 | ITGA2B HDAC10 H2BW2 AQP9 NCF2 H2BW1 H2BC11 PRKCG RAC2 FCGR2A H2BC5 H4C8 ITGAM FPR2 PIK3CD CLEC7A H3C13 H2BC13 FPR3 H2AC13 H2AC11 H3C12 CR1L H2BC12 FCGR3A H3C14 H2BC18 H2BC15 H4C15 H4C14 H4C12 H2BC14 H3C8 H2BC6 H2BC17 H2AC12 H4C13 H3C11 H2BC9 H3C1 H4C9 H2AC14 H2BC3 H2AC16 H4C5 H2AC8 H4C4 H3C7 H2AC4 H2BC10 H4C1 H2AC17 H4C2 H3C10 H3C2 H3C3 |
| Alcoholism | 2.985 | 6.50E-11 | 50 | CREB3L3 HDAC10 H2BW2 GRIN2D H2BW1 H2BC11 GNGT1 CAMK4 H2BC5 H4C8 GNG3 GNG8 TH H3C13 H2BC13 H2AC13 H2AC11 H3C12 H2BC12 H3C14 H2BC18 H2BC15 H4C15 H4C14 H4C12 H2BC14 H3C8 H2BC6 H2BC17 H2AC12 H4C13 H3C11 H2BC9 H3C1 H4C9 H2AC14 H2BC3 H2AC16 H4C5 H2AC8 H4C4 H3C7 H2AC4 H2BC10 H4C1 H2AC17 H4C2 H3C10 H3C2 H3C3 |
| Systemic lupus erythematosus | 3.858 | 3.27E-14 | 47 | H2BW2 IFNG C9 CD80 H2BW1 H2BC11 FCGR2A H2BC5 H4C8 H3C13 H2BC13 H2AC13 H2AC11 H3C12 H2BC12 FCGR3A H3C14 H2BC18 H2BC15 H4C15 H4C14 H4C12 H2BC14 H3C8 H2BC6 H2BC17 H2AC12 H4C13 H3C11 H2BC9 H3C1 H4C9 H2AC14 H2BC3 H2AC16 H4C5 H2AC8 H4C4 H3C7 H2AC4 H2BC10 H4C1 H2AC17 H4C2 H3C10 H3C2 H3C3 |
| Focal adhesion | 2.400 | 1.63E-06 | 43 | ITGA3 ITGA2B IBSP TNC LAMA3 LAMC2 RASGRF1 LAMB1 ITGA6 LAMA1 CAV1 MET MYL7 COL1A1 PDGFRB ITGB6 FN1 SPP1 PGF PRKCG RAC2 ITGB4 COL4A2 ITGA11 COL2A1 COL6A1 COL6A2 EGFR VEGFC VAV2 ITGA5 COL6A3 COL1A2 RAC3 PIK3CD THBS2 COL4A1 COL4A5 LAMB3 FLNA PDGFA COL4A6 ITGA1 |
| Viral carcinogenesis | 2.310 | 6.14E-06 | 42 | MAD1L1 EIF2AK2 TRAF1 CREB3L3 HDAC10 H2BW2 CCNE1 CDK6 CDC20 H2BW1 RBPJL H2BC11 CCNA1 CCNA2 CDKN2A CHEK1 H2BC5 H4C8 CDK1 PIK3CD CCNE2 CCR8 TBPL2 H2BC13 H2BC12 H2BC18 H2BC15 H4C15 H4C14 H4C12 H2BC14 H2BC6 H2BC17 H4C13 H2BC9 H4C9 H2BC3 H4C5 H4C4 H2BC10 H4C1 H4C2 |
| Regulation of actin cytoskeleton | 1.903 | 0.001683584 | 37 | ITGA3 ITGA2B INSRR PFN2 FGF4 ITGA6 MYL7 FGF8 PDGFRB ITGB6 FN1 RAC2 ARPC1B ITGB4 ITGA11 FGF5 DIAPH3 ITGAX GNA12 EGFR MSN ITGAD FGF17 VAV2 FGFR4 ITGA5 FGF19 RAC3 ITGAM PIK3CD F2 F2R IQGAP3 CHRM5 FGF3 PDGFA ITGA1 |
| Transcriptional misregulation in cancer | 2.035 | 0.000792787 | 35 | HOXA11 TRAF1 HOXA9 CEBPE GZMB MMP9 MET PLAU GRIA3 SSX1 CCNA1 CCNA2 IGFBP3 HMGA2 MMP3 CSF2 TLX3 CXCL8 ITGAM DDIT3 H3C13 WT1 PAX5 H3C12 PDGFA NTRK1 H3C14 HOXA10 H3C8 H3C11 H3C1 H3C7 H3C10 H3C2 H3C3 |
| ECM-receptor interaction | 4.186 | 2.76E-11 | 33 | ITGA3 ITGA2B IBSP TNC LAMA3 LAMC2 HMMR LAMB1 ITGA6 LAMA1 COL1A1 ITGB6 FN1 SPP1 ITGB4 COL4A2 ITGA11 COL2A1 COL6A1 COL6A2 DSPP DMP1 ITGA5 COL6A3 COL1A2 NPNT THBS2 COL4A1 COL4A5 AGRN LAMB3 COL4A6 ITGA1 |
| Calcium signaling pathway | 1.535 | 0.081969536 | 33 | CAMK1G MCOLN3 FGF4 NTSR1 GRIN2D MET FGF8 PDGFRB SLC8A2 PRKCG NGF ITPKA FGF5 EGFR HTR2C CACNA1B HTR7 VEGFC CYSLTR2 CAMK4 FGF17 FGFR4 PTGER1 TPCN2 DRD5 ADRA1B TRHR F2R CHRM5 FGF3 ADRB3 PDGFA NTRK1 |
| Protein digestion and absorption | 3.468 | 9.91E-09 | 32 | SLC7A9 COL11A1 COL17A1 COL5A3 COL16A1 SLC7A8 COL1A1 COL12A1 COL7A1 SLC8A2 COL10A1 COL5A1 COL4A2 COL2A1 COL6A1 COL6A2 COL8A1 SLC7A7 CPA2 COL26A1 COL6A3 COL1A2 COL3A1 COL22A1 COL24A1 SLC6A19 COL4A1 COL4A5 COL27A1 COL13A1 COL4A6 COL5A2 |
| Cell cycle | 2.835 | 1.60E-06 | 32 | MAD1L1 MCM2 SMC1B ORC1 ORC6 CDC45 CDC6 MCM5 CDC25B E2F1 MCM4 CCNE1 TGFB1 CDK6 TTK CDC20 PKMYT1 CCNA1 CCNB1 CCNA2 CDKN2A CHEK1 BUB1B CCNB2 CDC25C MAD2L1 PTTG1 MCM7 PLK1 BUB1 CDK1 CCNE2 |
| JAK-STAT signaling pathway | 2.067 | 0.001683584 | 30 | IL12RB2 IL11 IL12RB1 OSM IL21R IFNG PDGFRB STAT1 IL13RA2 EPO IL2RA STAT4 IL21 EGFR IFNK IL24 CSF2 IL22RA2 IL7R STAT2 PIK3CD IFNB1 IFNL1 IFNL2 IFNE SOCS1 IFNL3 PDGFA IFNA1 CSH2 |
| MicroRNAs in cancer | 2.011 | 0.003303846 | 29 | BRCA1 TNC PTGS2 FSCN1 DNMT3B MMP9 CDC25B E2F1 CCNE1 CDK6 MET PDGFRB STMN1 PLAU PRKCG HOXD10 KIF23 EGFR CDCA5 CDKN2A HMGA2 MMP16 IGF2BP1 ITGA5 PIK3CD CCNE2 SOCS1 PDGFA TRIM71 |
| Proteoglycans in cancer | 1.492 | 0.168475285 | 27 | PLAUR MMP9 TGFB1 CAV1 MET WNT2 COL1A1 NANOG FN1 WNT6 PLAU PRKCG HOXD10 WNT2B WNT10A LUM EGFR MSN WNT7A VAV2 ITGA5 COL1A2 FZD6 PIK3CD FZD2 WNT7B FLNA |
| Hepatitis C | 1.778 | 0.038747432 | 25 | EIF2AK2 CLDN18 E2F1 CDK6 OAS3 OAS2 IFNG STAT1 RSAD2 EGFR PPP2R2B MX1 CLDN14 CLDN19 FADD CXCL10 STAT2 CLDN20 PIK3CD IFNB1 MX2 CLDN6 IFIT1 CLDN24 IFNA1 |
