# Supplementary material for: A dual-functional antiviral and anticancer peptide to target intricate host-pathogen interacting proteins of human papillomavirus associated with head and neck cancer
Source: PLoS One. 2026 Oct 9;21(10):e0359513. doi: 10.1371/journal.pone.0359513 (PMC13652162; doi:10.1371/journal.pone.0359513)
Supplement: Supplementary file 2 [file pone.0359513.s002.docx]

**S2 Table Pathogen proteins involved in HNSCC**

| **Uniprot** | **Gene ID** | **Gene name** | **Organism** |
| --- | --- | --- | --- |
| P03126 | E6 | Protein E6 | Human papillomavirus type 16 |
| P03129 | E7 | Protein E7 | Human papillomavirus type 16 |
| P03101 | L1 | Major capsid protein L1 | Human papillomavirus type 16 |
| P03107 | L2 | Minor capsid protein L2 | Human papillomavirus type 16 |
| P06927 | E5 | Probable protein E5 | Human papillomavirus type 16 |
| P03120 | E2 | Regulatory protein E2 | Human papillomavirus type 16 |
| A0A089NDG7 | L1 | Major capsid protein L1 | Human papillomavirus type 16 |
| P06789 | E1 | Replication protein E1 | Human papillomavirus type 18 |
| P06788 | E7 | Protein E7 | Human papillomavirus type 18 |
| P06792 | E5 | Probable protein E5 | Human papillomavirus type 18 |
| P06463 | E6 | Protein E6 | Human papillomavirus type 18 |
| P06790 | E2 | Regulatory protein E2 | Human papillomavirus type 18 |
| P06429 | E7 | Protein E7 | Human papillomavirus 33 |
| P06427 | E6 | Protein E6 | Human papillomavirus 33 |
| P06423 | E2 | Regulatory protein E2 | Human papillomavirus 33 |
