# Supplementary material for: A dual-functional antiviral and anticancer peptide to target intricate host-pathogen interacting proteins of human papillomavirus associated with head and neck cancer
Source: PLoS One. 2026 Oct 9;21(10):e0359513. doi: 10.1371/journal.pone.0359513 (PMC13652162; doi:10.1371/journal.pone.0359513)
Supplement: Supplementary file 3 [file pone.0359513.s003.docx]

**S3 Table. Host proteins involved in HNSCC**

| **Uniprot ID** | **Gene ID** | **Gene name** | **LogFC** | **p-value** |
| --- | --- | --- | --- | --- |
| Q08117 | HOXC9 | Homeobox protein Hox-C9 | 3.633 | 3.8E-53 |
| P12272 | PTHLH | Parathyroid hormone-related protein | 3.407 | 1.49E-38 |
| P23510 | TNFSF4 | Tumor necrosis factor ligand superfamily member 4 | 2.726 | 3.59E-30 |
| Q16787 | LAMA3 | Laminin subunit alpha-3 | 2.722 | 3.98E-32 |
| O14746 | TERT | Telomerase reverse transcriptase | 2.692 | 8.59E-25 |
| Q14439 | GPR176 | G-protein coupled receptor 176 | 2.382 | 7.68E-32 |
| Q9UQ26 | RIMS2 | Regulating synaptic membrane exocytosis protein 2 | 2.290 | 1.17E-17 |
| Q15858 | SCN9A | Sodium channel protein type 9 subunit alpha | 2.213 | 6.53E-10 |
| P35520 | CBS | Cystathionine beta-synthase | 2.149 | 2.09E-10 |
| P09914 | IFIT1 | Interferon-induced protein with tetratricopeptide repeats 1 | 2.101 | 3.55E-16 |
| O75762 | TRPA1 | Transient receptor potential cation channel subfamily A member 1 | 2.057 | 1.26E-22 |
| Q14332 | FZD2 | Frizzled-2 | 1.993 | 7.08E-40 |
| P14174 | MIF | Macrophage migration inhibitory factor | 1.955 | 0.0076 |
| Q6UVK1 | CSPG4 | Chondroitin sulfate proteoglycan 4 | 1.854 | 5.35E-14 |
| Q08050 | FOXM1 | Forkhead box protein M1 | 1.830 | 3.07E-41 |
| P78396 | CCNA1 | Cyclin-A1 | 1.828 | 4.05E-14 |
| Q13158 | FADD | FAS-associated death domain protein | 1.767 | 4.72E-17 |
| P99999 | TK1 | Thymidine kinase, cytosolic | 1.763 | 2.51E-42 |
| Q03135 | CAV1 | Caveolin-1 | 1.699 | 1.02E-15 |
| P11166 | SLC2A1 | Solute carrier family 2, facilitated glucose transporter member 1 | 1.693 | 1.78E-22 |
| Q969G5 | CAVIN3 | Caveolae-associated protein 3 | 1.609 | 9.4E-18 |
| O94851 | MICAL2 | [F-actin]-monooxygenase MICAL2 | 1.595 | 1.56E-19 |
| P25787 | PSMA2 | Proteasome subunit alpha type-2 | 1.510 | 2.05E-17 |
| Q9UPY5 | SLC7A11 | Cystine/glutamate transporter | 1.491 | 0.0000002 |
| P50281 | MMP14 | Matrix metalloproteinase-14 | 1.443 | 4.61E-29 |
| P07093 | SERPINE2 | Glia-derived nexin | 1.368 | 4.28E-09 |
| P28300 | LOX | Protein-lysine 6-oxidase | 1.300 | 1.62E-09 |
| P16144 | ITGB4 | Integrin beta-4 | 1.279 | 6E-19 |
| P06493 | CDK1 | Cyclin-dependent kinase 1 | 1.267 | 1.07E-26 |
| Q01860 | POU5F1 | POU domain, class 5, transcription factor 1 | 1.251 | 5.34E-11 |
| Q03518 | TAP1 | Antigen peptide transporter 1 | 1.239 | 7.88E-13 |
| P17936 | IGFBP3 | Insulin-like growth factor-binding protein 3 | 1.236 | 2.99E-08 |
| P09619 | PDGFRB | Platelet-derived growth factor receptor beta | 1.166 | 2.06E-09 |
| P17302 | GJA1 | Gap junction alpha-1 protein | 1.154 | 7.53E-09 |
| P52292 | KPNA2 | Importin subunit alpha-1 | 1.145 | 2.31E-33 |
| Q9GZM7 | TINAGL1 | Tubulointerstitial nephritis antigen-like | 1.128 | 2.43E-08 |
| P20248 | CCNA2 | Cyclin-A2 | 1.121 | 7.31E-19 |
| P25116 | F2R | Proteinase-activated receptor 1 | 1.103 | 6.51E-12 |
| Q8WUM9 | SLC20A1 | Sodium-dependent phosphate transporter 1 | 1.102 | 1.57E-23 |
| Q92597 | NDRG1 | Protein NDRG1 | 1.078 | 3.6E-09 |
| P38398 | BRCA1 | Breast cancer type 1 susceptibility protein | 1.067 | 4.49E-21 |
| P30532 | CHRNA5 | Neuronal acetylcholine receptor subunit alpha-5 | 1.049 | 5.95E-11 |
| P35080 | PFN2 | Profilin-2 | 1.049 | 6.21E-08 |
| P18462 | HLA-A | HLA class I histocompatibility antigen, A alpha chain | 1.039 | 1.67E-11 |
| P00533 | EGFR | Epidermal growth factor receptor | 1.034 | 0.0000005 |
| P21333 | FLNA | Filamin-A | 1.029 | 2.35E-13 |
| O60353 | FZD6 | Frizzled-6 | 1.024 | 7.9E-17 |
| O43586 | PSTPIP1 | Proline-serine-threonine phosphatase-interacting protein 1 | 1.017 | 3.83E-09 |
