# Supplementary material for: A dual-functional antiviral and anticancer peptide to target intricate host-pathogen interacting proteins of human papillomavirus associated with head and neck cancer
Source: PLoS One. 2026 Oct 9;21(10):e0359513. doi: 10.1371/journal.pone.0359513 (PMC13652162; doi:10.1371/journal.pone.0359513)
Supplement: Supplementary file 4 [file pone.0359513.s004.docx]

**S4 Table. Host protein structure with Ramachandran favoured regions**

| **Protein** | **Initial Energy** | **Final Energy** | **Ramachandran favoured region (%)** | **Ramachandran plot** | **Protein structure** |
| --- | --- | --- | --- | --- | --- |
| CAV1 | -4698.77 | -6376.92 | 100 |  |  |
| CCNA2 | -8490.57 | -12029.37 | 100 |  |  |
| CDK1 | -13087.17 | -15708.41 | 99.6 |  |  |
