# Supplementary material for: A dual-functional antiviral and anticancer peptide to target intricate host-pathogen interacting proteins of human papillomavirus associated with head and neck cancer
Source: PLoS One. 2026 Oct 9;21(10):e0359513. doi: 10.1371/journal.pone.0359513 (PMC13652162; doi:10.1371/journal.pone.0359513)
Supplement: Supplementary file 5 [file pone.0359513.s005.docx]

**S5 Table. Viral protein structure with Ramachandran favoured regions**

| **Protein** | **Initial Energy** | **Final Energy** | **Ramachandran favoured region (%)** | **Ramachandran plot** | **Protein structure** |
| --- | --- | --- | --- | --- | --- |
| E5_HPV16 | -2020.04 | -3435.75 | 100 |  |  |
| E6_HPV33 | -7939.59 | -10209.13 | 100 |  |  |
| E7_HPV16 | -2011.33 | -3322.72 | 98.8 |  |  |
| E7_PHV18 | -3506.62 | -4997.89 | 98.9 |  |  |
| E7_PHV33 | -3035.22 | -4272.62 | 98.8 |  |  |
