# Supplementary material for: A dual-functional antiviral and anticancer peptide to target intricate host-pathogen interacting proteins of human papillomavirus associated with head and neck cancer
Source: PLoS One. 2026 Oct 9;21(10):e0359513. doi: 10.1371/journal.pone.0359513 (PMC13652162; doi:10.1371/journal.pone.0359513)
Supplement: Supplementary file 6 [file pone.0359513.s007.docx]

**S7 Table. Secondary structure analyses of the screened peptides**

| **Peptides** | **Length** | **Alpha helix** | **Beta turn** | **Random coil** | **Extended strand** | **Ramachandran favours** | **Ramachandran Plot** | **Modelled Peptide** |
| --- | --- | --- | --- | --- | --- | --- | --- | --- |
| Peptide 1 | 43 | 62.79% | 9.30% | 27.91% | - | 100 |  |  |
| Peptide 2 | 40 | 40.00% | 10.00% | 42.50% | 7.50% | 100 |  |  |
| Peptide 3 | 25 | 88.00% | - | 12.00% | - | 100 |  |  |
| Peptide 4 | 30 | 60.00% | - | 23.33% | 16.67% | 100 |  |  |
| Pep11** (Control) | 19 | - | 52.63% | - | 47.37% | 100 |  |  |
| Pep-7  (Control) | 12 | - | 83.33% | 16.67% | - | 100 |  |  |
