# Supplementary material for: A dual-functional antiviral and anticancer peptide to target intricate host-pathogen interacting proteins of human papillomavirus associated with head and neck cancer
Source: PLoS One. 2026 Oct 9;21(10):e0359513. doi: 10.1371/journal.pone.0359513 (PMC13652162; doi:10.1371/journal.pone.0359513)
Supplement: Supplementary file 7 [file pone.0359513.s008.docx]

**S8 Table Docking validation of Lead Peptide 4 and two controls against all the target proteins through HDOCK**

|  | **Pep11** (Control)** | | **Pep-7 (Control)** | | **Peptide 4 (Lead Peptide from ClusPro-based docking)** | |
| --- | --- | --- | --- | --- | --- | --- |
| **Cancer and Viral Target Proteins** | **Docking Score** | **Confidence score** | **Docking Score** | **Confidence score** | **Docking Score** | **Confidence score** |
| **CAV1** | -203.59 | 0.7449 | -169.26 | 0.5951 | **-330.32** | 0.9736 |
| **CCNA2** | -178.94 | 0.6408 | -155.8 | 0.529 | **-226.94** | 0.8233 |
| **CDK1** | -213.7 | 0.7814 | -175.44 | 0.6245 | **-218.2** | 0.7964 |
| **E5_16** | -181.37 | 0.6519 | -181.89 | 0.6543 | **-275.05** | 0.9242 |
| **E6_33** | -203.82 | 0.7458 | -169.84 | 0.5979 | **-258.02** | 0.8966 |
| **E7_16** | -149.48 | 0.4974 | -143.73 | 0.4687 | **-221.62** | 0.8073 |
| **E7_18** | -172.14 | 0.6089 | -155.89 | 0.5294 | **-203.13** | 0.7432 |
| **E7_33** | -151.75 | 0.5087 | -152.81 | 0.514 | **-221.76** | 0.8077 |
