# Supplementary material for: A dual-functional antiviral and anticancer peptide to target intricate host-pathogen interacting proteins of human papillomavirus associated with head and neck cancer
Source: PLoS One. 2026 Oct 9;21(10):e0359513. doi: 10.1371/journal.pone.0359513 (PMC13652162; doi:10.1371/journal.pone.0359513)
Supplement: Supplementary file 8 [file pone.0359513.s009.docx]

**S9 Table. Molecular dynamics simulation analysis on 3 different Host apo-proteins and their complex with Peptide 4**

|  | **Unit** | **ApoProtein CAV1** | **CAV1-Pep4** | **ApoProtein CCNA2** | **CCNA2-Pep4** | **ApoProtein CDK1** | **CDK1-Pep4** |
| --- | --- | --- | --- | --- | --- | --- | --- |
| **Average RMSD** | nm | 2.0278 ± 0.36 | 1.8749 ± 0.4 | 0.2223 ± 0.02 | 0.2088 ± 0.01 | 0.2146 ± 0.02 | 0.179 ± 0.02 |
| **Average RMSF** | nm | 0.7904 ± 0.27 | 0.6772 ± 0.33 | 0.114 ± 0.05 | 0.4708 ± 0.13 | 0.0999 ± 0.04 | 0.0958 ± 0.04 |
| **Average Rg** | nm | 2.1683 ± 0.27 | 2.3288 ± 0.2 | 1.8543 ± 0.01 | 1.8392 ± 0.01 | 1.9726 ± 0.01 | 2.0359 ± 0.01 |
| **Average SASA** | nm^2^ | 106.6339 ± 1.35 | 125.3957 ± 1.33 | 147.1947 ± 1.23 | 165.5966 ± 1.37 | 168.7045 ± 1.32 | 183.898 ± 1.62 |
| **H bond** | Max H bonds | N/A | 21 | N/A | 16 | N/A | 15 |
|  | Most Constant | N/A | 11 | N/A | 10 | N/A | 8 |
| **Interaction energy** | kj/mol | N/A | -490.086 | N/A | -296.044 | N/A | -392.148 |
