# Supplementary material for: A dual-functional antiviral and anticancer peptide to target intricate host-pathogen interacting proteins of human papillomavirus associated with head and neck cancer
Source: PLoS One. 2026 Oct 9;21(10):e0359513. doi: 10.1371/journal.pone.0359513 (PMC13652162; doi:10.1371/journal.pone.0359513)
Supplement: Supplementary file 9 [file pone.0359513.s010.docx]

**S10 Table. Molecular dynamics simulation analysis on 5 different HPV (Pathogen) apo-proteins and their complex with Peptide 4**

|  | **Unit** | **ApoProtein E5_16** | **E5_16-Pep4** | **ApoProtein E6_33** | **E6_33-Pep4** | **ApoProtein E7_16** | **E7_16-Pep4** | **ApoProtein E7_18** | **E7_18-Pep4** | **ApoProtein E7_33** | **E7_33-Pep4** |
| --- | --- | --- | --- | --- | --- | --- | --- | --- | --- | --- | --- |
| **Average RMSD** | nm | 0.8053 ± 0.2 | 0.39962 ± 0.07 | 0.4596 ± 0.1 | 0.4561 ± 0.05 | 0.5706 ± 0.1 | 0.4676 ± 0.08 | 0.9116 ± 0.2 | 0.6359 ± 0.1 | 0.8229 ± 0.1 | 0.6982 ± 0.06 |
| **Average RMSF** | nm | 0.3388 ± 0.16 | 0.1909 ± 0.1 | 0.2512 ± 0.18 | 0.1994 ± 0.16 | 0.4014 ± 0.18 | 0.2281 ± 0.1 | 0.4998 ± 0.14 | 0.2798 ± 0.18 | 0.4467 ± 0.20 | 0.2806 ± 0.11 |
| **Average Rg** | nm | 1.2673 ± 0.1 | 1.4715 ± 0.01 | 1.7296 ± 0.02 | 1.7912 ± 0.02 | 1.7054 ± 0.1 | 1.6885 ± 0.02 | 1.7109 ± 0.08 | 1.7652 ± 0.04 | 1.5943 ± 0.07 | 1.6359 ± 0.04 |
| **Average SASA** | nm^2^ | 49.38325 ± 0.99 | 66.77 ± 1.08 | 84.9859 ± 1.50 | 103.1337 ± 1.23 | 61.1254 ± 1.11 | 78.8987 ± 1.01 | 65.0365 ± 1.17 | 83.6427 ± 1.15 | 61.712 ±1.09 | 79.9693 ± 0.99 |
| **H bond** | Max H bonds | N/A | 6 | N/A | 15 | N/A | 22 | N/A | 23 | N/A | 15 |
|  | Most Constant | N/A | 1 | N/A | 6 | N/A | 14 | N/A | 17 | N/A | 8 |
| **Interaction energy** | kj/mol | N/A | -421.355 | N/A | -271.429 | N/A | -289.942 | N/A | -383.134 | N/A | -185.237 |
