# Supplementary material for: A dual-functional antiviral and anticancer peptide to target intricate host-pathogen interacting proteins of human papillomavirus associated with head and neck cancer
Source: PLoS One. 2026 Oct 9;21(10):e0359513. doi: 10.1371/journal.pone.0359513 (PMC13652162; doi:10.1371/journal.pone.0359513)
Supplement: Supplementary file 10 [file pone.0359513.s011.docx]

**S1 Figure RMSD comparison of all complexes through 100 ns MD Simulation replicate run**
